# Metabolism of F18, a Derivative of Calanolide A, in Human Liver Microsomes and Cytosol

**DOI:** 10.3389/fphar.2017.00479

**Published:** 2017-07-19

**Authors:** Xiangmeng Wu, Qinghao Zhang, Jiamei Guo, Yufei Jia, Ziqian Zhang, Manman Zhao, Yakun Yang, Baolian Wang, Jinping Hu, Li Sheng, Yan Li

**Affiliations:** ^1^State Key Laboratory of Bioactive Substance and Function of Natural Medicines, Beijing Laboratory of Non-Clinical Drug Metabolism and PK/PD Study, Key Laboratory of Active Substances Discovery and Drug Ability Evaluation, Department of Drug Metabolism, Institute of Materia Medica, Chinese Academy of Medical Sciences & Peking Union Medical College Beijing, China; ^2^Beijing Key Laboratory of New Drug Mechanisms and Pharmacological Evaluation Study, Institute of Materia Medica, Chinese Academy of Medical Sciences & Peking Union Medical College Beijing, China

**Keywords:** calanolide A, human immunodeficiency virus, drug metabolism, cytochrome P450s, flavin-containing monooxygenase, carbonyl reductase

## Abstract

10-Chloromethyl-11-demethyl-12-oxo-calanolide (F18), an analog of calanolide A, is a novel potent nonnucleoside reverse transcriptase inhibitor against HIV-1. Here, we report the metabolic profile and the results of associated biochemical studies of F18 *in vitro* and *in vivo*. The metabolites of F18 were identified based on liquid chromatography-electrospray ionization mass spectrometry and/or nuclear magnetic resonance. Twenty-three metabolites of F18 were observed in liver microsomes *in vitro*. The metabolism of F18 involved 4-propyl chain oxidation, 10-chloromethyl oxidative dechlorination and 12-carbonyl reduction. Three metabolites (M1, M3-1, and M3-2) were also found in rat blood after oral administration of F18 and the reduction metabolites M3-1 and M3-2 were found to exhibit high potency for the inhibition of HIV-1 *in vitro*. The oxidative metabolism of F18 was mainly catalyzed by cytochrome P450 3A4 in human microsomes, whereas flavin-containing monooxygenases and 11β-hydroxysteroid dehydrogenase were found to be involved in its carbonyl reduction. In human cytosol, multiple carbonyl reductases, including aldo-keto reductase 1C, short-chain dehydrogenases/reductases and quinone oxidoreductase 1, were demonstrated to be responsible for F18 carbonyl reduction. In conclusion, the *in vitro* metabolism of F18 involves multiple drug metabolizing enzymes, and several metabolites exhibited anti-HIV-1 activities. Notably, the described results provide the first demonstration of the capability of FMOs for carbonyl reduction.

## Introduction

HIV infection is a major cause of life-threatening conditions and leads to the development of AIDS. Approximately 36.9 million people worldwide are living with HIV, and approximately 2 million people were newly infected in 2014 (Gubernick et al., 2016). Despite considerable efforts, no effective regimen to eliminate the virus is currently available. Combination antiretroviral therapy is the standard treatment for patients infected with HIV and consists of two nucleoside reverse transcriptase (RT) inhibitors combined with a nonnucleoside RT inhibitor (NNRTI), a protease inhibitor, or a integrase inhibitor (Gunthard et al., 2016). However, due to the error-prone nature of HIV RT and the lack of a proofreading function, resistance to antiretroviral drugs is generally unavoidable (Roberts et al., 1988). Therefore, until a cure for this disease is found, new inhibitors with high activity and improved safety are urgently needed.

**Figure 1:**
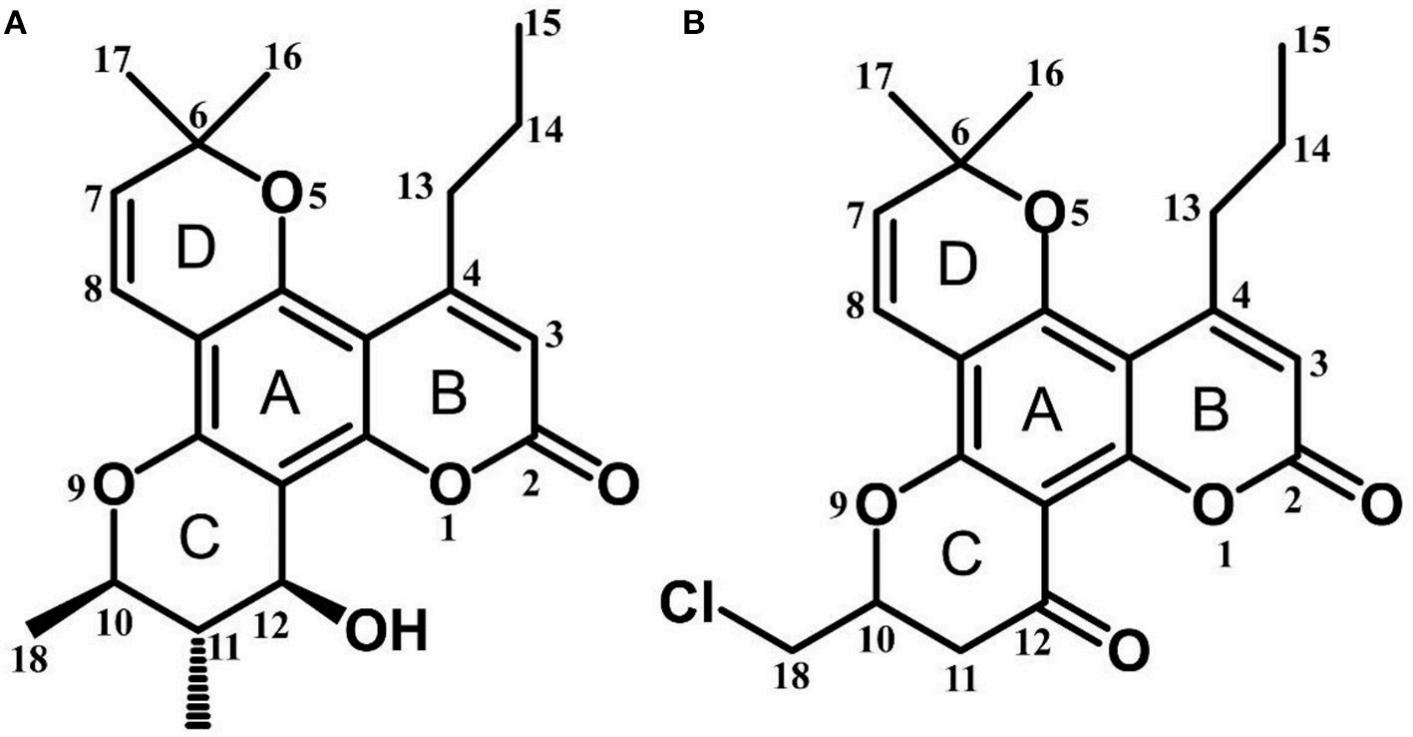
Chemical structures of calanolide A **(A)** and F18 **(B)**.

Calanolide A (Figure 1A, C_22_H_26_O_5_, MW = 370), a natural product originally extracted from a tropical tree (*Calophyllum lanigerum*), has been identified as an attractive NNRTI against HIV-1, particularly resistant virus strains (Currens et al., 1996a; Buckheit et al., 1999; Quan et al., 1999). Unlike conventional NNRTIs, calanolide A has been postulated to compete with deoxynucleoside triphosphates for binding to the HIV-1 RT active site (Currens et al., 1996b). Phase I studies have found that calanolide A is well tolerated. Consequently, it has potential clinical applications in combination with other antiviral drugs to suppress HIV-1 mutants (Creagh et al., 2001; Eiznhamer et al., 2002). Nevertheless, the development of calanolide A has been delayed due to its low therapeutic index (range: 16–279), non-ideal antiviral activity, and the complexity of its extraction from plants (Currens et al., 1996a; Flavin et al., 1996). Thus, a structural optimization of calanolide A was performed to identify novel analogs with high potency against HIV-1. Zembower et al. (1997) illustrated that the 12-ketone form of calanolide exhibits anti-HIV-1 activity; however, this analog possesses a lower therapy index than its parent natural product calanolide A. Ma et al. (2008) found that 10-bromomethyl-12-oxo-calanolide A has significantly enhanced antiviral potency, while a bromine atom may confer toxicity.

10-Chloromethyl-11-demethyl-12-oxo-calanolide A (F18, Figure 1B) is a novel analog of calanolide A, and unlike calanolide A, F18 has a low half-maximal effective concentration (EC_50_) (7.4 nM) and a high therapeutic (1417). Notably, F18 presents extremely high potency against the clinical Y181C single mutation of HIV-1 (Xue et al., 2010). Lu et al. (2012) systematically investigated the pharmacology of F18 and concluded that F18 might be an attractive antiretroviral candidate for clinical regimens aiming to treat HIV-1-infected patients.

Despite extensive investigations of its pharmacological activities and mechanisms, the biotransformation of F18 has not yet been fully elaborated. The determination of drug metabolic stability and its primary metabolic pathways and the characterization of the enzyme involved in the drug metabolism are major facets of the drug discovery and development continuum (Foti and Dalvie, 2016). Moreover, many researchers have studied the chemical modifications of calanolide A and related natural species to identify new analogs with higher anti-HIV potency (Galinis et al., 1996; Sekino et al., 2004; Ma et al., 2008; Xue et al., 2010). To the best of our knowledge, the metabolic characterizations of calanolide A analogs have not been described in the literature. Because F18 is an analog of calanolide A, the metabolic characterization of F18 might facilitate the development of calanolide A analogs for the treatment of HIV-1 infection. Therefore, the objectives of the present study were as follows: (1) to identify the major metabolites of F18 and the enzyme(s) responsible for F18 metabolism *in vitro* and (2) to evaluate the anti-HIV activities of major metabolites of F18 and their exposure in rats after oral administration.

## Materials and methods

### Cell, plasmids, and chemicals

Human embryonic kidney (HEK) 293T cells were purchased from the China Infrastructure of Cell Line Resources (Beijing, China). The pNL4-3.Luc.R-E plasmid was obtained from the National Institute of Health AIDS Research and Reference Reagent Program. The vesicular stomatitis virus glycoprotein (VSV-G) plasmid was a gift from Dr. Lijun Rong (University of Illinois, Chicago, IL, USA). F18 (racemic mixture, purity > 99%) was provided by Prof. Gang Liu (Department of Medicinal Chemistry, Institute of Materia Medica, Chinese Academy of Medical Science & Peking Union Medical College, Beijing, China). NADH, β-NADP, glucose-6-phosphate (6-G-P), 6-G-P dehydrogenase (6-G-P-DH), propranolol, furafylline, tranylcypromine, ticlopidine, quercetin, sulfaphenazole, quinidine, diethyldithiocarbamate, ketoconazole, benzydamine, aminobenzotriazole (ABT), 18β-glycyrrhetinic acid, 2-chloroethyl ethyl sulfide (2-CEE), phenobarbital, flufenamic acid, chenodeoxycholic, 4-methylpyrazole, menadione, medroxyprogesterone, and dicumarol were obtained from Sigma-Aldrich (St. Louis, MO, USA). High-performance liquid chromatography (HPLC)-grade methanol and acetonitrile were obtained from Merck (Darmstadt, Germany). Analytical-grade diethyl ether was purchased from Beijing Chemical Reagent Co., Ltd. (Beijing, China). Purified water was generated with a Milli-Q Gradient system (Millipore Corporation, Molsheim, France). *d*_6_*-*Dimethyl sulfoxide (*d*_6_-DMSO) was obtained from Cambridge Isotope Laboratories, Inc. (Andover, MA, USA).

### Animals and enzymes

Male Sprague-Dawley rats (200–220 g, 6–8 weeks) were obtained from Beijing Vital River Experimental Animal Co., Ltd. (Beijing, China). Rat liver microsomes (RLMs) were prepared by differential ultracentrifugation. In brief, male SD rats were sacrificed after starvation for 12 h. The thoracic cavity was then opened, and the liver was perfused *in situ* with 0.9% NaCl. The liver was excised and homogenized with three volumes of TMS buffer (50-mM Tris, 5-mM MgCl_2_, and 200-mM sucrose, pH 7.4). The homogenate was centrifuged at 10,000 × *g* and 4°C for 20 min, decant the supernatant and centrifuged again at 105,000 × *g* for 60 min. The pellet of microsomes was resuspended with TMS buffer. The protein concentration was determined through bicinchoninic acid (BCA) assay (Beyotime Institute of Biotechnology, Jiangsu, China). Pooled mixed-gender human liver microsomes (HLMs), male beagle dog liver microsomes (DLMs), male monkey liver microsomes (MLMs), pooled mixed-gender human liver cytosol (HLcy), recombinant human cytochrome P450 enzymes (CYP1A1, CYP1A2, CYP2A6, CYP2B6, CYP2C8, CYP2C9, CYP2C19, CYP2D6, CYP2E1, CYP2J2, CYP3A4, CYP4F2, CYP4F3, and CYP4A11), and recombinant human flavin-containing monooxygenases (FMO1, FMO3, and FMO5) were purchased from BD Gentest (Woburn, MA, USA).

The animals were permitted full access to standard laboratory food and water and were maintained on a 12-h light/dark cycle in air-conditioned animal quarters at room temperature (25 ± 2°C) with 50 ± 5% relative humidity. The rats were placed on a fasting regimen 12 h before the experiment with free access to water. All animal protocols were approved by the Animal Care and Welfare Committee of Institute of Materia Medica, Chinese Academy of Medical Sciences & Peking Union Medical College (Beijing, China). In addition, all animal experiments were conducted in strict accordance with the guidelines for the care and use of laboratory animals issued by the Institute Animal Care and Welfare Committee.

### Incubation of F18 with liver microsomes and cytosols

A stock solution of F18 (5 mM) was prepared in methanol. F18 (50 μM) was incubated with RLMs/DLMs/MLMs/HLMs and HLcy (0.5 mg protein/mL) in 50 mM Tris-HCl buffer containing 5 mM MgCl_2_ (pH 7.4) in a final volume of 200 μL. The final concentration of methanol in the incubation system was 1% (v/v). After 3 min of preincubation at 37°C, the reactions were initiated by the addition of NADPH-regenerating system (10-mM β-NADP, 100-mM 6-G-P and 10-U/mL 6-G-P-DH) or NADH. After incubation for 1 h, the reactions were terminated by the addition of two volumes of ice-cold acetonitrile. Samples without NADPH or NADH were included as controls. The incubation mixture was vortexed and centrifuged at 13,300 × *g* for 5 min. A 10 μL aliquot of the supernatant was injected into an LC-tandem mass spectrometer (LC-MS/MS) for analysis.

### Preparation and isolation of F18 metabolites in microsomal incubations

For preparation of the major metabolites, F18 (100 μM) was incubated with HLMs (0.5 mg protein/mL) or RLMs (1 mg protein/mL) in Tris-HCl buffer as described above. After preincubation at 37°C for 3 min, an NADPH-generating system was added, and the mixture was incubated for 1 h. The total incubation volume was 200 mL. Diethyl ether (600 mL) was added to terminate the enzymatic reaction, and the mixture was vigorously shaken and maintained at 37°C for 2 h. The organic phase was collected and evaporated to dryness in a water bath at 40°C. The residue obtained was reconstituted with 1 mL of acetonitrile and injected into an Agilent 1100 HPLC system equipped with a quaternary pump and an ultraviolet (UV) diode array detector (DAD) (Agilent Technologies, Santa Clara, CA, USA) for further purification.

Chromatographic separation was performed on a reverse-phase semi-preparative column (9.4 × 250 mm i.d., Zorbax, 5 μm) with a C8 guard column (9.4 × 15 mm, 5 μm) using mobile phases of acetonitrile (mobile phase A), methanol (mobile phase B) and water (mobile phase C) at a flow rate of 2 mL/min, and the separation was performed in two steps. The samples were first fractionated using elution condition 1 (Table 1), yielding four fractions (F1–F4). Fractions F2 and F4 were then further purified using elution conditions 2 and 3, respectively (Table 1). F18 and its metabolites were measured at 265 nm. Fraction F2 afforded M1 (0.8 mg), M5-2 (1.8 mg), M5-3 (1.7 mg), M5-4 (1.5 mg), and M5-5 (1.5 mg), whereas Fraction F4 afforded M2-9 (0.8 mg), M3-1 (6.1 mg), and M3-2 (2.5 mg).

**Table 1:** Elution conditions for the preparation of F18 metabolites.

**1**			**2**			**3**			
**Time (min)**	**Mobile phase**		**Time (min)**	**Mobile phase**		**Time (min)**	**Mobile phase**		
	**A**	**C**		**A**	**C**		**A**	**B**	**C**
0	55	45	0	32	68	0	60	8	32
10	55	45	15	32	68	40	60	8	32
30	30	70	35	28	72	50	58	8	34
35	20	80	36	10	90	70	54	8	38
45	10	90	41	10	90	71	2	8	90
50	10	90	42	32	68	76	2	8	90
52	55	45	60	32	68	77	60	8	32
65	55	45				100	60	8	32

Condition **1** was used to elute the incubated sample and yielding four fractions (F1-F4);

Condition **2** was used to elute fraction F2 and afford M1, M5-2, M5-3, M5-4, and M5-5;

*Condition **3** was used to elute fraction F4 and afford M2-9, M3-1, and M3-2*.

*A, Acetonitrile; B, methanol; C, water*.

### Nuclear magnetic resonance analysis

F18 and its metabolites were dissolved in *d*_6_-DMSO. ^1^H nuclear magnetic resonance (NMR) spectra were collected at 600 MHz using an Agilent NMR vnmrs600 spectrometer (Agilent Technologies, Santa Clara, CA, USA), and the chemical shifts were recorded in parts per million referenced to the solvent signal. Rotating-frame nuclear overhauser effect correlation spectroscopy (ROESY) spectra of M3-1 and M3-2 were also obtained to determine their relative configuration.

### Identification of the human enzymes responsible for the oxidative metabolism of F18 in HLMs

#### Microsomal incubations of F18 in the presence of chemical inhibitors of CYPs or thermal inactivation

The contributions of CYPs and FMOs to the metabolism of F18 were investigated by chemical inhibition and thermal inactivation, respectively. Selective inhibitors of CYPs and their concentrations were selected based on the Food and Drug Administration's (FDA's) *Drug Development and Drug Interactions* (Supplemental Table S1) https://www.fda.gov/drugs/developmentapprovalprocess/developmentresources/druginteractionslabeling/ucm093664.htm, as follows: ABT (1 mM) for broad CYPs, furafylline (10 μM) for CYP1A2, tranylcypromine (2 μM) for CYP2A6, ticlopidine (0.5 μM) for CYP2B6, quercetin (10 μM) for CYP2C8, sulfaphenazole (5 μM) for CYP2C9, ticlopidine (10 μM) for 2C19, quinidine (2 μM) for CYP2D6, diethyldithiocarbamate (50 μM) for CYP2E1, and ketoconazole (1 μM) for CYP3A4. Briefly, F18 (10 μM) was incubated with HLMs (0.5 mg protein/mL) and an NADPH-generating system in the absence and presence of CYP inhibitors at 37°C for 30 min, and the reaction was terminated with two volumes of ice-cold acetonitrile. The thermal inactivation of FMOs was conducted as reported by Ziegler (1988). In brief, HLMs (0.5 mg protein/mL) were preheated at 50°C for 5 min and then immersed in an ice-cold bath. After the addition of F18 (10 μM) and equilibration for 3 min at 37°C, the reaction was started by the addition of the NADPH-generating system. After a 30-min incubation, the reactions were terminated as described above. Benzydamine (an FMO substrate) was used as the positive control for FMO activity. The retained activity of CYPs was confirmed using midazolam (a CYP3A4 substrate). The formation of the major metabolites M1, M2-9, M3-1, M3-2, M5-2, M5-3, M5-4, and M5-5 was analyzed by LC-MS/MS.

#### Metabolism of F18 by cDNA-expressed human enzymes

To further verify the roles of CYPs and FMOs in the metabolism of F18 in humans, F18 (10 μM) was incubated individually with 14 cDNA-expressed human CYP isozymes (50-pmol/mL CYP1A1, CYP1A2, CYP2A6, CYP2B6, CYP2C8, CYP2C9, CYP2C19, CYP2D6, CYP2E1, CYP2J2, CYP3A4, CYP4F2, CYP4F3, and CYP4A11) and three cDNA-expressed FMO enzymes (50-pmol/mL FMO1, FMO3, and FMO5). The incubation conditions were similar to those used for HLMs (described above), with the exception of the incubating time (1 h). Samples incubated without NADPH were used as the negative control.

### Identification of the human enzymes responsible for the carbonyl reduction of F18 in HLMs and HLcy

Selective chemical inhibitors were used to investigate the enzymes responsible for the carbonyl reduction of F18 in both HLMs and HLcy. Two concentrations of each chemical inhibitor were selected based on their 50% inhibitory concentration (IC_50_) or *Ki* values reported for specific carbonyl reductases. The carbonyl-reducing enzymes, their selective inhibitors and the concentrations used were as follows: 11β-hydroxysteroid dehydrogenase (11β-HSD), 18β-glycyrrhetinic acid (0.1 and 0.5 μM); NADPH-CYP reductase, 2-CEE (1 and 5 mM); aldo-keto reductases (AKRs), phenobarbital flufenamic acid, chenodeoxycholic acid and medroxyprogesterone (10 and 50 μM); short-chain dehydrogenases/reductases (SDRs), menadione and quercetin (10 and 50 μM); quinone oxidoreductase 1 (NQO1), dicumarol (10 and 50 μM); and alcohol dehydrogenases (ADHs), 4-methylpyrazole (100 and 500 μM). F18 (10 μM) was incubated with HLMs (0.5 mg/mL) and the NADPH-generating system for 30 min or with HLcy and a cofactor (NADPH or NADH) for 10 min in the presence of chemical inhibitors under the above-described conditions. Control incubations using the vehicles but not the chemical inhibitors were also performed. The formation of M3-1 and M3-2 was quantified by LC-MS/MS.

### Pharmacokinetic studies

To investigate the pharmacokinetic characteristics of F18 and its major metabolites, five rats were intragastrically administered F18 at a dose of 50 mg/kg. Blood samples were collected in heparinized ice-bath tubes from the suborbital veniplex at 0, 0.25, 0.5, 1, 2, 4, 6, 8, 12, 16, and 24 h postdose. The blood concentrations of F18 and its major metabolites were determined by LC-MS/MS and their pharmacokinetic parameters were calculated through non-compartmental analysis using WinNonlin Version 6.1 (Pharsight, Mountain View, CA, USA).

### Anti-HIV-1 activity of F18 and its metabolites *In vitro*

The metabolites M1, M2-9, M3-1, M3-2, M5-2, M5-3, M5-4, and M5-5 were screened for their activities against HIV-1 using pseudotyped viruses as described previously (Ma et al., 2013). Briefly, VSV-G/HIV-1 viruses were prepared by co-transfecting 293T cells with VSV-G plasmid and env-deficient HIV-1 vector (pNL4-3.luc.R-E-, Supplemental Figures S1, S2). The virions were quantified by measuring the p24 levels using an enzyme-linked immunosorbent assay (ELISA) kit (ZeptoMetrix, Cat. 0801111) with a dilution of 0.2 ng p24/mL.

One day before infection, 293T cells were seeded at a density of 6 × 10^4^ cells/well in 24-well plates and then infected with VSV-G/HIV-1 viruses in the presence or absence of the tested compounds. The infected cells were lysed 48 h post-infection using a cell lysis reagent (Promega), and the luciferase activity of the cell lysate was measured with a Sirius luminometer (Berthold Detection System). For comparison, the anti-HIV-1 activity of F18 was evaluated under the same conditions.

### LC-MS/MS

Metabolic profiling studies were performed with a Finnigan TSQ Quantum MS instrument with an electrospray ionization probe and a Finnigan SURVEYOR HPLC system (Thermo Finnigan, San Jose, CA, USA). HPLC was performed with a Zorbax SB-C18 column (2.1 × 100 mm, 3.5 μm, Agilent Corporation, CA, USA) with a column temperature of 35°C and a flow rate of 200 μL/min. F18 and its metabolites were eluted using a linear gradient method with a mobile phase consisting of solvent A (water with 0.1% formic acid) and solvent B (acetonitrile with 0.1% formic acid). The gradient used was as follows: Solvent B was maintained at 20% for 5 min, linearly increased to 70% over 45 min, rapidly increased to 90% in 1 min, maintained at 90% for 9 min, decreased to 20% over 1 min and maintained at 20% for 6 min. The samples were analyzed in the positive-ionization mode, and the TSQ capillary temperature was set to 350°C. Nitrogen was used as both the sheath gas and the auxiliary gas and was delivered at 30 psi and 15 psi, respectively. The spray voltage and tube lens were set to 3,800 V and 123 V, respectively. The full-width half maximum (FWHM) was set to 0.7. MS data were acquired in the selective ion-recording mode or by collecting full scans from 200 to 600 *m/z* at 1 scan/s. The mass spectrometer and HPLC system were operated using Xcalibur 2.0.7 software.

The amounts of F18, M1, M2-9, M3-1, M3-2, M5-2, M5-3, M5-4, and M5-5 in the incubations were simultaneously quantified with the LC-MS/MS system. The HPLC system was the same as that used for the metabolic profiling studies described above, with the exception of the elution gradient, which was as follows: Solvent B was maintained at 15% for 2 min, rapidly increased to 35% in 1 min, increased to 45% over 27 min, increased to 65% in 5 min, slowly increased to 67% over 10 min, increased to 90% in 1 min, maintained at 90% for 4 min, decreased to 15% in 1 min and maintained at 15% for 7 min. For the blood samples, F18, M1, M3-1, and M3-2 were simultaneously quantified using the following elution gradient: Solvent B was maintained at 15% for 2 min, increased to 85% in 1 min, maintained at 85% for 5 min, decreased to 15% in 0.5 min and maintained at 15% for 4.5 min. Detection was also performed using a Finnigan TSQ Quantum MS instrument with an electrospray ionization probe operated in the positive mode. The sheath gas, auxiliary gas, spray voltage and tube lens parameters were the same as those for the metabolic profiling studies. Selective reaction monitoring (SRM) was used for the quantification of each compound, and the specific transitions monitored were *m/z* 388.8→360.8 (22 V) for F18, *m/z* 260.0→183.0 (17 V) for the internal standard (IS, propranolol), *m/z* 370.9→342.9 (22 V) for M1, *m/z* 386.9→358.9 (22 V) for M2-9, *m/z* 390.9→372.8 (20 V) for M3-1 and M3-2, and *m/z* 404.8→358.7 (22 V) for M5-2, M5-3, M5-4 and M5-5.

Calibration curves for F18 and its metabolites were fitted via linearly weighted (1/x^2^) least-squares regression. The correlation coefficient values of F18 and its metabolites in the incubations were >0.99 for the range 10–5,000 nM; the LOQ was 10 nM with a signal-to-noise ratio >10:1; the accuracies were 90–113% and the precisions (RSD) were <10.5%. In the blood samples, the correlation coefficient values for F18, M1, M3-1, and M3-2 were >0.99 for the range 5–2,000 nM; the LOQ was 5 nM with the signal-to-noise ratio >10:1; the accuracies were 91–112% and the precisions (RSD) were <9.6%. In addition, no interference was found in the retention time of each analyte in both the incubated samples and the blood samples.

## Results

### Metabolites of F18 *In vitro*

**Figure 2:**
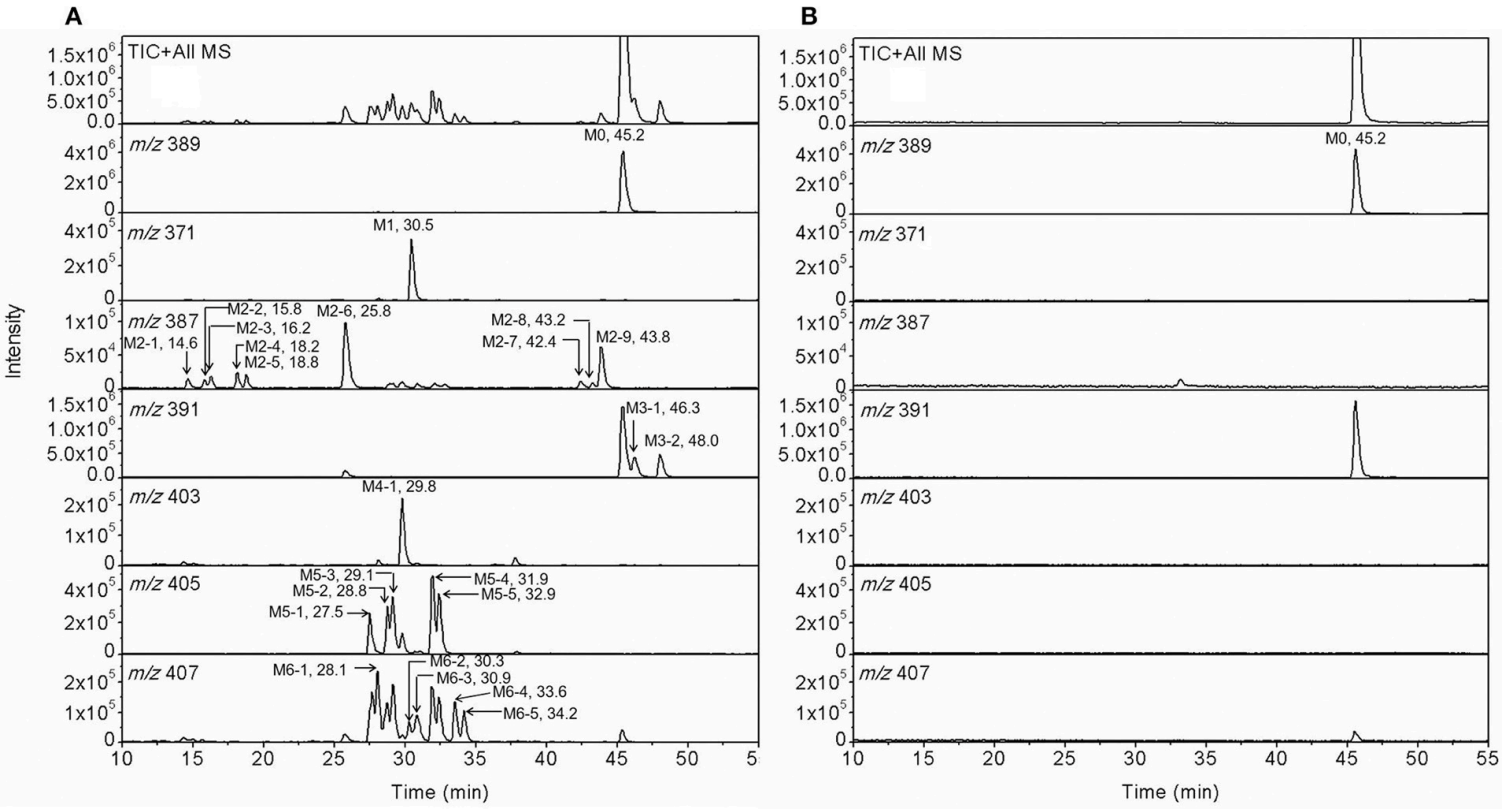
Representative extracting ion [M+H]^+^ chromatograms of F18 and its metabolites in HLM incubation with **(A)** and without NADPH-regenerating system **(B)**. The 23 detected metabolites are labeled M1 (*m/z* 371), M2 (*m/z* 387), M3 (*m/z* 391), M4 (*m/z* 403), M5 (*m/z* 405), and M6 (*m/z* 407), and the parent compound is labeled M0 (*m/z* 389). The metabolites were numbered according to their chromatographic retention times.

For the metabolic profiling of F18, the supernatant extracted from the incubation of F18 with HLMs was monitored by LC-MS/MS in the full-scan mode (Figure 2), and 23 phase I metabolites were detected. Based on the selected ion, F18 metabolites can be classified into six types: oxidative dechlorination metabolites-M1 (*m/z* 371), dehydrogenation metabolites-M2 (*m/z* 387), hydrogenation metabolites-M3 (*m/z* 391), hydroxylation and dehydrogenation metabolites-M4 (*m/z* 403), hydroxylation metabolites-M5 (*m/z* 405) and hydroxylation and hydrogenation metabolites-M6 (*m/z* 407). These metabolites were further numbered according to their chromatographic retention times. The phase I metabolite profiles of F18 in RLMs, DLMs, and MLMs were similar to those in HLMs (Table 2, Supplemental Figure S3), although the metabolic sites exhibited slight discrepancies among the experimental liver microsomes. The hydrogenation metabolites M3-1 and M3-2 were also generated in HLcy in the presence of NADPH or NADH.

**Table 2:** F18 metabolites detected in HLM, RLM, DLM, MLM, and HLcy after incubation with a substrate concentration of 50 μM for 1 h.

**Metabolite**	**m/z**	**description**	**Retention time (min)**	**HLM**	**RLM**	**DLM**	**MLM**	**HLcy with NADPH**	**HLcy with NADH**
M1	371	+OH−Cl	30.5	+	+	+	+	−	−
M2-1	387	−2H	14.6	+	−	−	+	−	−
M2-2	387	−2H	15.8	+	−	+	+	−	−
M2-3	387	−2H	16.2	+	−	+	+	−	−
M2-4	387	−2H	18.2	+	−	−	+	−	−
M2-5	387	−2H	18.8	+	−	−	+	−	−
M2-6	387	−2H	25.8	+	+	+	+	−	−
M2-7	387	−2H	42.4	+	+	−	−	−	−
M2-8	387	−2H	43.2	+	+	−	−	−	−
M2-9	387	−2H	43.8	+	+	−	−	−	−
M3-1	391	+2H	46.3	+	+	+	+	+	+
M3-2	391	+2H	48.0	+	+	+	+	+	+
M4-1	403	+O−2H	29.8	+	+	+	+	−	−
M5-1	405	+O	27.5	+	+	−	+	−	−
M5-2	405	+O	28.8	+	+	+	+	−	−
M5-3	405	+O	29.1	+	+	+	+	−	−
M5-4	405	+O	31.9	+	+	−	+	−	−
M5-5	405	+O	32.9	+	+	−	+	−	−
M6-1	407	+O+2H	28.1	+	+	−	+	−	−
M6-2	407	+O+2H	30.3	+	+	+	+	−	−
M6-3	407	+O+2H	30.9	+	+	+	+	−	−
M6-4	407	+O+2H	33.6	+	+	−	+	−	−
M6-5	407	+O+2H	34.2	+	+	−	+	−	−

*+, Metabolite detected; −, metabolite not detected*.

*HLM, Human liver microsomes; RLM, rat liver microsomes; DLM, dog liver microsomes; MLM, monkey liver microsomes; HLcy, human liver cytosol*.

### Structural identification of F18 metabolites in HLMs by LC-MS/MS and NMR

**Figure 3:**
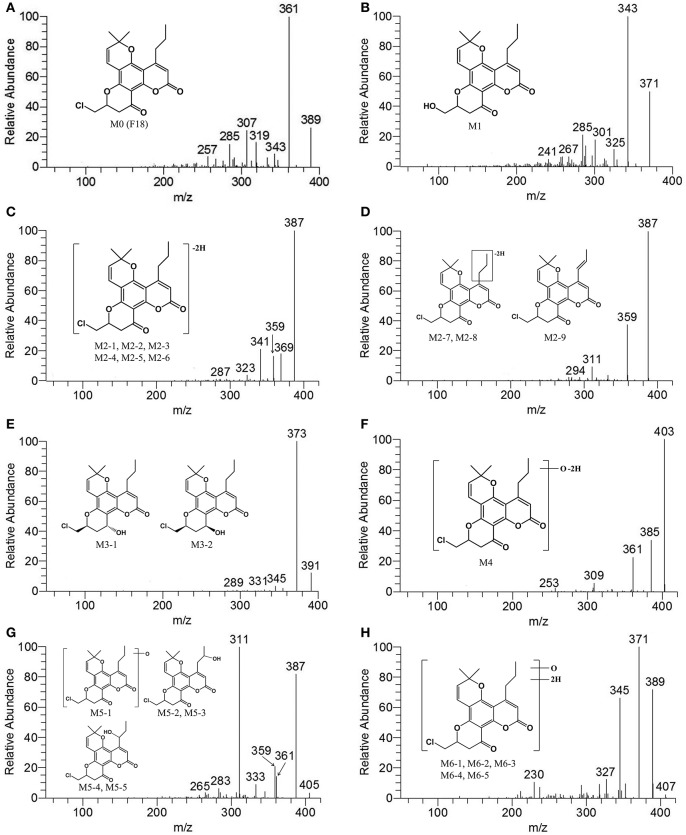
MS^2^ spectra of F18 and its metabolites in HLM incubation in the presence of a NADPH-generating system. **(A)**, F18 (*m/z* 389); **(B)**, M1 (*m/z* 371); **(C)**, M2-1, M2-2, M2-3, M2-4, M2-5, and M2-6 (*m/z* 387); **(D)**, M2-7, M2-8, and M2-9 (*m/z* 387); **(E)**, M3-1 and M3-2 (*m/z* 391); **(F)**, M4 (*m/z* 403); **(G)**, M5-1, M5-2, M5-3, M5-4, and M5-5 (*m/z* 405); and **(H)**, M6-1, M6-2, M6-3, M6-4, and M6-5 (*m/z* 407). MS^2^ data were obtained from the respective [M+H]^+^ ions as the precursors for collision-induced dissociation.

A comprehensive understanding of the fragmentation behavior of the parent compound is very helpful in metabolite identification using LC-MS/MS. The protonated F18 molecule (*m/z* 389) was detected in the positive-ion mode and further dissociated in MS^2^ to produce fragment ions at *m/z* 361 [(M+H-CO)^+^], 343 [(M+H-CO-H_2_O)^+^], 319 [(M+H-CO-C_3_H^6^)^+^], 307 [(M+H-CO-3H_2_O)^+^], and 285 [(M+H-CO-C_3_H_5_Cl)^+^] (Figure 3A). The adduct ions of F18 were also observed at *m/z* 411 [(M+Na)^+^].

Structural identification of the metabolites was achieved by interpreting the mass shifts in the MS/MS spectra relative to that of the parent compound. The proposed fragmentation patterns of the metabolites are shown in Figure 3. To explicitly determine the structures of major metabolites, eight metabolites were isolated from the human/RLM incubation, and their structures were further characterized by NMR. The ^1^H NMR data are provided in Table 3 and Supplemental Figure S4.

Table 3^1^H-NMR spectra data for F18 and its major metabolites.

**Table:** 

**Position**	**M0**	**M1**	**M2-9**	**M3-1**	**M3-2**
3	6.129 (1H, s)	6.117 (1H, s)	6.157 (1H, s)	6.006 (1H, s)	6.023 (1H, s)
7	5.836 (1H, d, 10.2)	5.806 (1H, d, 10.2)	6.597 (1H, d, 10.2)	6.566 (1H, d, 10.2)	6.578 (1H, d, 10.2)
8	6.608 (1H, d, 10.2)	6.699 (1H, d, 10.2)	5.841 (1H, d, 10.2)	5.719 (1H, d, 10.2)	5.725 (1H, d, 10.2)
10	4.894 (1H, m)	4.598 (1H, m)	4.902 (1H, m)	4.557 (1H, m)	4.64 (1H, m)
11	Ha 2.832-2.917 (1H, m)	Ha 2.811 (1H, dd, 16.2, 12.6)	Ha 2.9 (1H, dd, 16.2, 12.6)	Ha 2.012 (1H, m)	Ha 2.202 (1H, m)
	Hb 2.671 (1H, dd, 16.2, 3)	Hb 2.556 (1H, dd, 16.2, 3)	Hb 2.673 (1H, dd, 16.2, 3)	Hb 1.77 (1H, m)	Hb 2.114 (1H, m)
12	–	–	–	4.983 (1H, s)	4.969 (1H, t, 4.2)
12-OH	–	–	–	5.372 (1H, d, 4.2)	5.304 (1H, s)
13	2.832-2.917 (2H, m)	2.87 (2H, m)	7.042 (1H, d, 15)	2.788-2.91 (2H, m)	2.785-2.937 (2H, m)
14	1.558 (2H, m)	1.577 (2H, m)	6.208 (1H, m)	1.574 (2H, m)	1.574 (2H, m)
15	0.979 (3H, t, 7.2)	0.982 (3H, t, 7.2)	1.89 (3H, d 6)	0.978 (3H, t, 7.2)	0.985 (3H, t, 7.2)
16,17	1.518, 1.477 (6H, 2s)	1.512, 1.479 (6H, 2s)	1.505, 1.464 (6H, 2s)	1.436, 1.424 (6H, 2s)	1.462, 1.435 (6H, 2s)
18	Ha 4.078 (1H,dd, 12, 3)	3.65-3.75 (2H, m)	Ha 4.073 (1H, m)	Ha 4.079 (1H,dd, 12, 3)	Ha 4.049 (1H,dd, 12, 9)
	Hb 3.983 (1H, dd, 12, 6)		Hb 3.983 (1H, dd, 12, 6)	Hb 3.956 (1H, dd, 12, 6)	Hb 3.929 (1H, dd, 12, 4.2)
18-OH	–	5.157 (1H, t, 6)	–	–	–
Structure	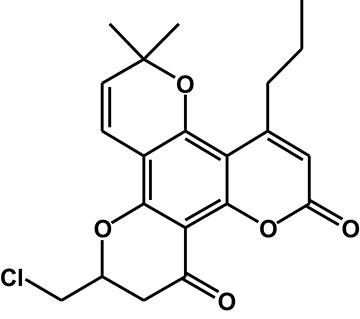	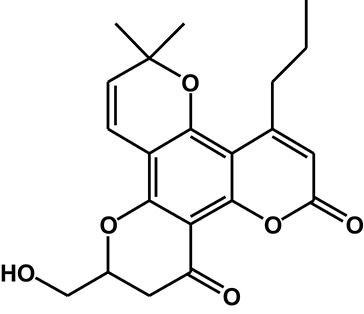	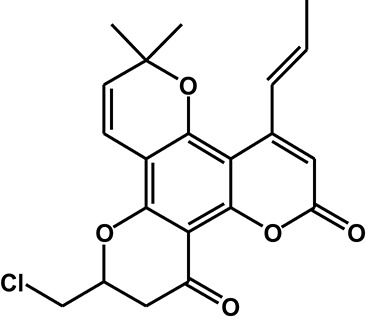	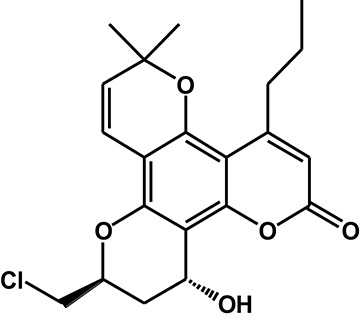	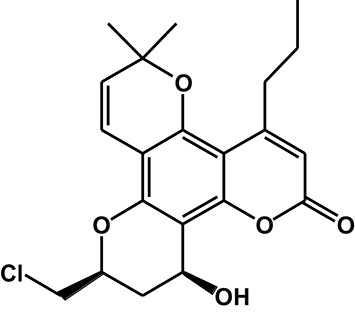

**Table:** 

**Position**	**M5-2**	**M5-3**	**M5-4**	**M5-5**
3	6.072 (1H, s)	6.074 (1H, s)	6.503 (1H, s)	6.50 (1H, s)
7	5.834 (1H, d, 10.2)	5.838 (1H, d, 10.2)	5.852 (1H, d, 10.2)	5.845 (1H, d, 10.2)
8	6.612 (1H, d, 10.2)	6.617 (1H, d, 10.2)	6.627 (1H, d, 10.2)	6.622 (1H, d, 10.2)
10	4.906 (1H, m)	4.885 (1H, m)	4.899 (1H, m)	4.918 (1H, m)
11	Ha 2.894 (1H, dd, 16.2, 12.6)	Ha 2.905(1H, dd, 16.2, 12.6)	Ha 2.916 (1H, dd, 16.2, 12.6)	Ha 2.896 (1H, dd, 16.2, 12.6)
	Hb 2.685 (1H, dd, 16.2, 3)	Hb 2.671 (1H, dd, 16.2, 3)	Hb 2.672 (1H, dd, 16.2, 3)	Hb 2.687 (1H, dd, 16.2, 3)
13	Ha 3.172 (1H, dd, 12.6, 4.2)	Ha 3.194 (1H, dd, 12.6, 4.2)	5.268 (1H, m)	5.247 (1H, m)
	Hb 2.712 (1H, m)	Hb 2.694 (1H, dd, 13.2, 4.2)		
13-OH	–	–	5.418 (1H, d, 4.8)	5.419 (1H, d, 4.8)
14	3.897 (1H, m)	3.87 (1H, m)	Ha 1.77 (1H, m)	Ha 1.801 (1H, m)
			Hb 1.256 (1H, m)	Hb 1.26 (1H, m)
14-OH	4.598 (1H, d, 5.4)	4.588 (1H, d, 5.4)	–	–
15	1.18 (3H, d, 6)	1.178 (3H, d, 6)	1.004 (3H, t, 7.2)	1.027 (3H, t, 7.2)
16,17	1.514, 1.468 (6H, 2s)	1.528, 1.471 (6H, 2s)	1.552, 1.465 (6H, 2s)	1.506, 1.516 (6H, 2s)
18	Ha 4.08 (1H, dd, 12, 3)	Ha 4.085 (1H, dd, 12, 3)	Ha 4.086 (1H, dd, 12, 3)	Ha 4.080 (1H, dd, 12, 3)
	Hb 3.982 (1H, dd, 12, 6)	Hb 3.987 (1H, dd, 12, 6)	Hb 3.988 (1H, dd, 12, 6)	Hb 3.983 (1H, dd, 12, 6)
				
Structure	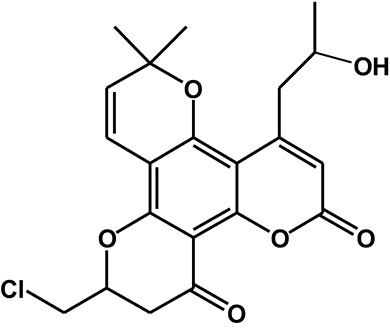	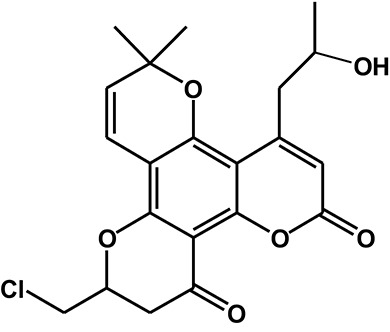	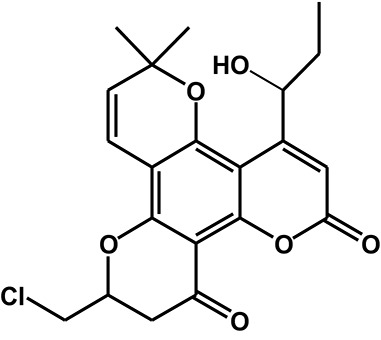	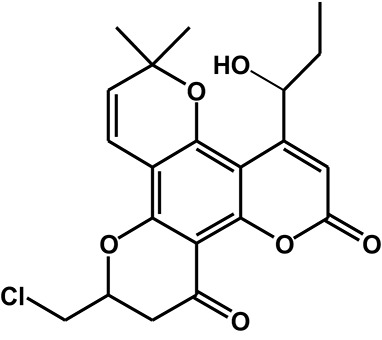

*Compounds were dissolved in d_6_-DMSO for NMR analysis on an Agilent NMR vnmrs600 spectrometer with ^1^H observed at 600 MHz. The chemical shifts were expressed in δ (ppm) relative to the solvent signal with coupling constants J in Hz. s, Singlet; d, doublet; t, triplet; m, multiplet*.

#### Metabolite M1

After HLM incubation, only one metabolite with a protonated molecular weight of 371 was detected. M1 and its fragment ions at *m/z* 343 [(M+H-CO)^+^], 325 [(M+H-CO-H_2_O)^+^], 301 [(M+H-CO-C_3_H^6^)^+^], and 289 [(M+H-CO-3H_2_O)^+^] were 18 Da smaller than those of the parent drug (Figure 3B). Because F18 contains one chlorine atom, the absence of an isotopic peak (M+H+2) reflected the loss of a chlorine atom from the parent drug. Thus, M1 can be identified as the oxidative dechlorination product of F18. This conclusion was supported by the ^1^H NMR spectral data (Table 3). Indeed, compared with the NMR spectra of F18, the proton signals of CH-10 (δ_H_ 4.60, m, 1H) and CH_2_-18 (δ_H_ 3.65–3.75, m, 2H) were shifted downfield, and proton signals corresponding to reactive hydrogen were observed, confirming that Cl was displaced by OH.

#### Metabolite M2

Up to nine metabolites with a protonated molecular weight of 387 were observed after HLM incubation, indicating that these metabolites were the dehydrogenation products of F18 (*m/z* 389). The metabolites can be divided into polar metabolites (M2-1, M2-2, M2-3, M2-4, M2-5, and M2-6) and less polar metabolites (M2-7, M2-8, and M2-9) based on their retention times. The MS/MS spectra (Figures 3C,D) revealed that the polar metabolites had the same fragmentation pattern with *m/z* 369 [(M+H-H_2_O)^+^], 359 [(M+H-CO)^+^] and 341 [(M+H-CO-H_2_O)^+^], while the less polar metabolites also exhibited the same fragment ions pattern with *m/z* 359 [(M+H-CO)^+^] and 311 [(M+H-C_3_H_5_Cl)^+^] indicating their structures similarity, respectively.

The structure of M2-9 was further characterized by NMR because it was present at a relatively high amount. The ^1^H NMR spectrum (Table 3) of M2-9 exhibited two additional peaks at δ_H_ 6.21 and 7.04 compared with that of F18, indicating that a new double bond must have formed in the metabolite. Further analysis of the ^1^H NMR spectra revealed that the proton signals of CH_2_-13 (δ_H_ 2.83–2.92, m, 2H) and CH_2_-14 (δ_H_ 1.56, m, 2H) disappeared and that the proton signals of CH_3_-15 shifted downfield from 0.98 to 1.89. These differences suggested that the double bond is located at C13 and C14 and confirmed that M2-9 is the 13,14-dehydrogenation product of F18. Dehydrogenation of M2-7 and M2-8 might also occur at the 4-propyl side chain. Moreover, dehydrogenation might also occur in the skeleton of the other metabolites (M2-1, M2-2, M2-3, M2-4, M2-5, and M2-6).

#### Metabolite M3

After HLM incubation, two metabolites (M3-1 and M3-2) afforded a protonated molecular peak at 391, which is 2 Da higher than that of the protonated parent drug, indicating the introduction of two hydrogen atoms. The MS/MS spectra of M3-1 and M3-2 (Figure 3E) showed identical fragment ions at 373 [(M+H-H_2_O)^+^] and 345 [(M+H-CO-H_2_O)^+^]. The structures of M3-1 and M3-2 were further characterized by NMR and the spectral data are presented in Table 3. A comparison of the ^1^H NMR spectra of M3-1 with that of F18 revealed the appearance of a reactive hydrogen signal at δ_H_ 5.30, suggesting that a carbonyl was replaced by an OH group. Moreover, the proton signals of CH-10 (δ_H_ 4.56, m, 1H) and CH_2_-11 [(δ_Ha_ 2.01, m, 1H), (δ_Hb_, 1.77 m, 1H)] were shifted upfield, indicating that the OH group was added to C13. The unchanged signals in the ^1^H NMR spectra of M3-1 compared with that of F18 support this hypothesis. Therefore, M3-1 was confirmed to be the 12-carbonyl reduction product of F18. A comparison of their ^1^H NMR spectra revealed similarities between M3-2 and M3-1, and the results indicated that these two metabolites share the same framework and planar structure. M3-2 differs from M3-1 only in the configuration at C-12. To determine the relative configurations of the epimers, ROESY spectra were obtained and a clear correlation was found between H-12 and H-10, which indicated that they are oriented on the same face in the structure of M3-2 (data not shown). Thus, M3-1 and M3-2 were identified as trans-12-hydroxy F18 and cis-12-hydroxy F18, respectively.

#### Metabolite M4

Metabolite M4 had a pronated molecular weight of 403, indicating the introduction of one oxygen atom with dehydrogenation. The isotopic peak (M+H+2) could also be detected. The MS/MS spectra (Figure 3F) showed fragment ions at *m/z* 385 [(M+H-H_2_O)^+^] and 361 [(M+H-C_3_H_6_)^+^]. M4 was tentatively identified as the monooxygenation and dehydrogenation product of F18.

#### Metabolite M5

Five metabolites with a protonated molecular weight of 405 were observed, suggesting that these metabolites are the hydroxylation products of F18. The MS/MS spectra (Figure 3G) show fragment ions at *m/z* 387 [(M+H-H_2_O)^+^], 361 [(M+H-CO_2_)^+^], 359 [(M+H-CO-H_2_O)^+^] and 311 [(M+H-C_3_H_5_Cl-H_2_O)^+^]. M5-1 was tentatively identified as the hydroxylation product of F18, although the position of the hydroxyl moiety was unclear. The structures of M5-2, M5-3, M5-4, and M5-5 were further characterized by NMR, and their ^1^H NMR spectral data are listed in Table 3. Compared with the ^1^H NMR spectra of F18, the skeleton proton signals of M5-2 were unchanged, suggesting that the skeleton was unmodified. Thus, the hydroxyl group might be attached to the 10-chloromethyl or 4-propyl moiety. Furthermore, the proton signals of CH_3_-15(δ_H_ 1.18, t, *J* = 6 Hz, 3H) and CH_2_-13 [(δ_Ha_ 3.17, dd, *J* = 12.6, 4.2 Hz, 1H), (δ_Hb_ 2.71, m, 1H)] were shifted downfield, and the CH_2_-14 methylene signal (δ_H_ 1.56, m, 2H) of F18 was replaced by a methenyl group (δ_H_ 3.90, m 1H), suggesting that the hydroxyl was attached to C-14. The ^1^H NMR spectral data of M5-3 were similar to those of M5-2, indicating that these two metabolites share the same planar structure. M5-3 differed from M5-2 only in its configuration at C-14. Accordingly, the C-14 epimers M5-2 and M5-3 were confirmed to be the 14-hydroxylation products of F18.

A comparison of the ^1^H NMR spectra of M5-4 and M5-5 with that of F18 revealed that the proton signals of CH_2_-14 [(δ_Ha_ 1.77, m, 1H), (δ_Hb_ 1.26, m, 1H)] were shifted downfield. Additionally, the CH_2_-13 methyl signal (δ_H_ 2.83–2.92, m, 2H) of F18 was shifted downfield and replaced by CH (δ_H_ 5.27, m, 1H). Therefore, M5-4 and M5-5 were confirmed to be the 13-hydroxylation products of F18. The only difference between these two metabolites was their configuration at C-13.

#### Metabolite M6

Five metabolites exhibited a protonated molecular ion peak at *m/z* 407, suggesting the introduction of an oxygen atom and two hydrogen atoms to the parent molecule. The MS/MS spectra (Figure 3H) showed identical fragment ions at *m/z* 389 [(M+H-H_2_O)^+^], 371 [(M+H-2H_2_O)^+^], and 345 [(M+H-CO_2_-H_2_O)^+^]. Due to their low relative amounts, M6-1, M6-2, M6-3, M6-4, and M6-5 were tentatively confirmed to be the hydroxyl and dehydrogenation products of F18.

### Identification of the metabolizing enzymes responsible for F18 metabolism in HLMs and HLcy

#### Identification of the enzymes that catalyze the oxidation of F18

**Figure 4:**
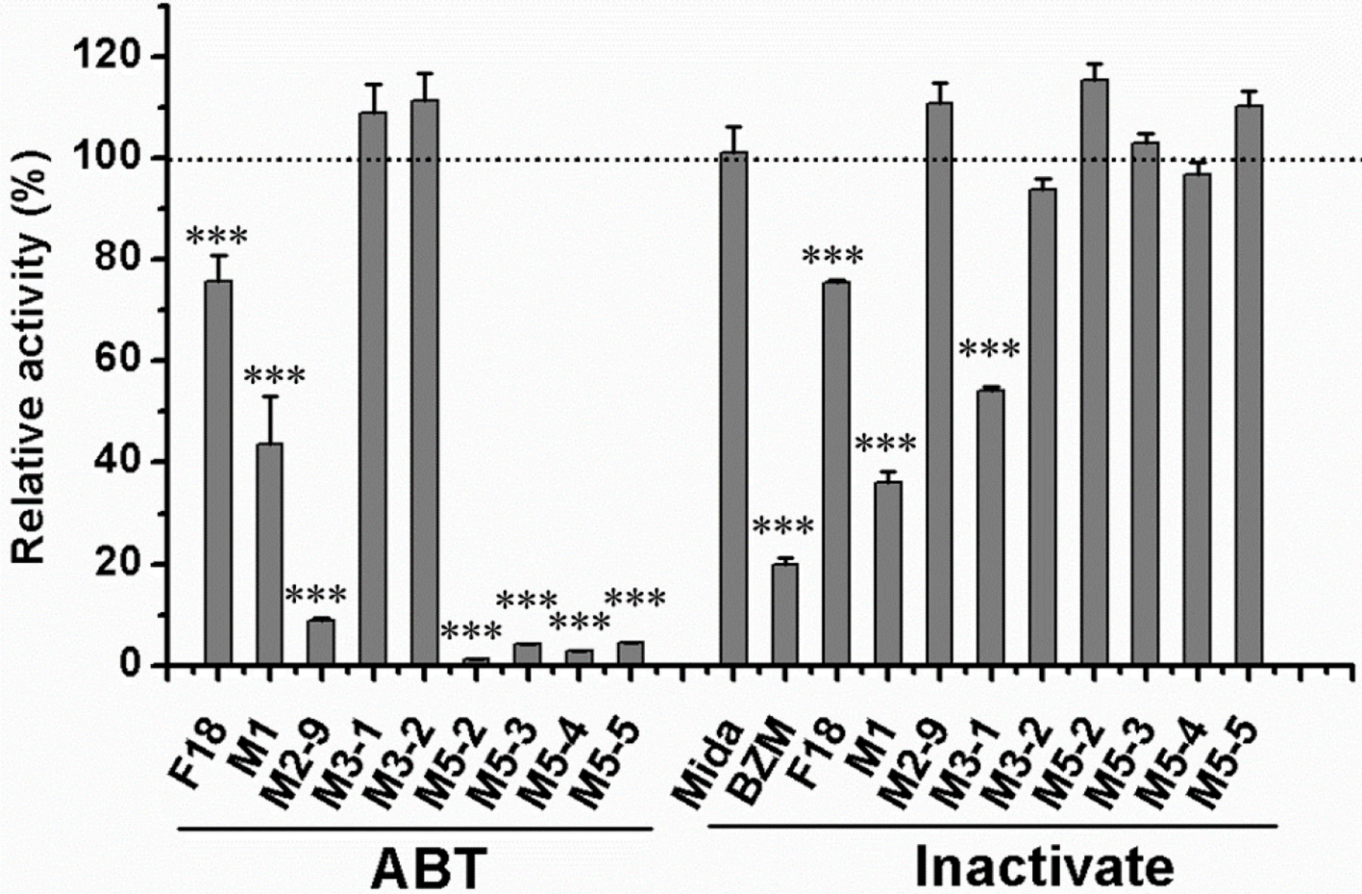
Effects of ABT and thermal inactivation on the metabolism of F18 in human liver microsomes. F18 (10 μM) was incubated with HLMs in the absence or presence of aminobenzotriazole for 30 min. For thermal inactivation studies, HLMs (0.5 mg protein/mL) were preincubated at 50°C for 5 min and then incubated with F18 (10 μM) for 10 min. The data are expressed as percentages of the control and represent the means ± SD from three independent experiments. BZM, Benzydamine; Mida, midazolam; ABT, aminobenzotriazole. Statistical significance was assessed using one-tailed student's *t*-test and is indicated with ^***^p < 0.005.

**Figure 5:**
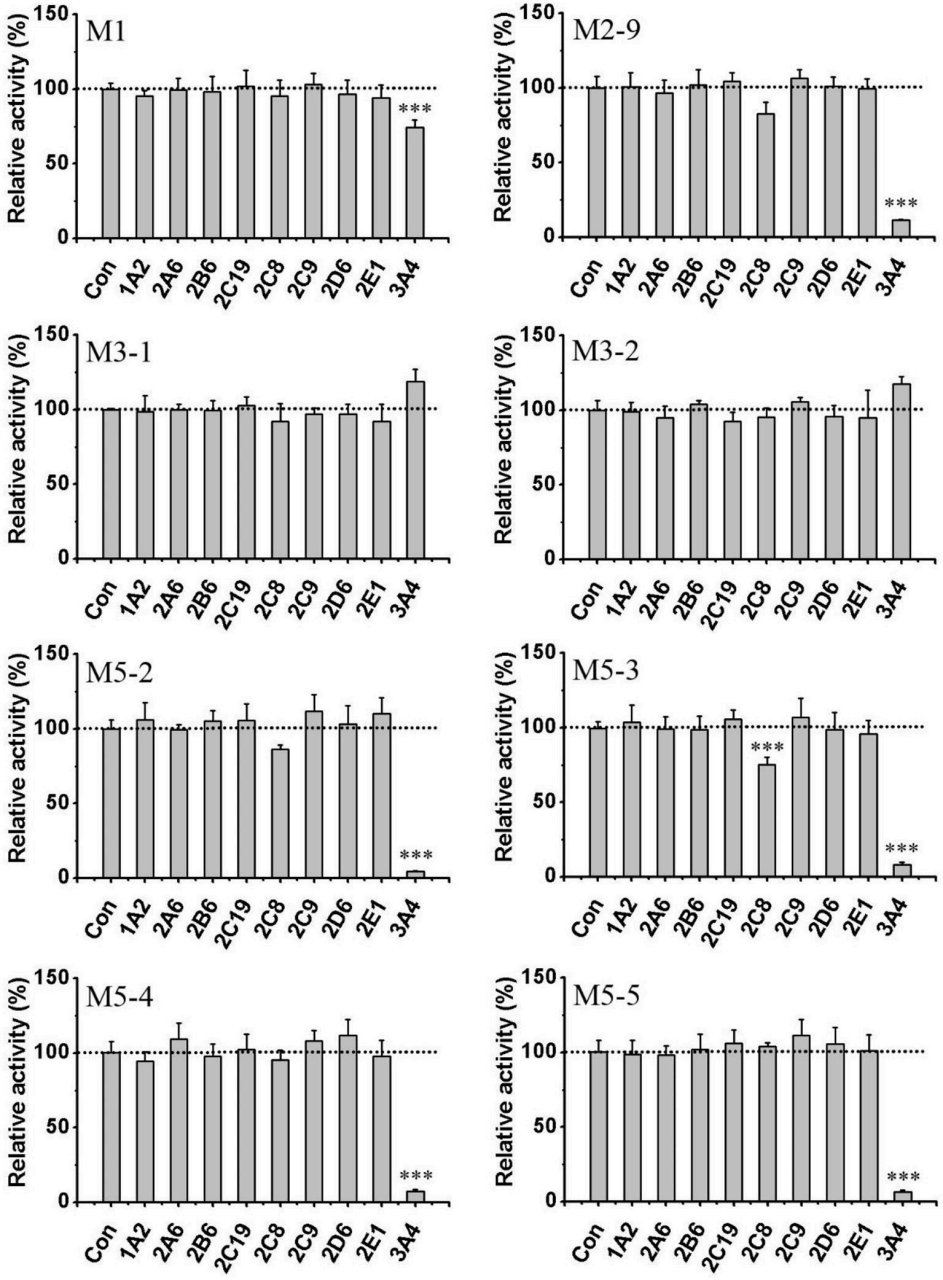
Effects of CYP450 inhibitors on the formation of F18 metabolites in HLMs. F18 (10 μM) was incubated with liver microsomes in the absence (as control) or presence of selective inhibitors: furafylline (10 μM) for CYP1A2, tranylcypromine (2 μM) for CYP2A6, ticlopidine (0.5 μM) for CYP2B6, quercetin (10 μM) for CYP2C8, sulfaphenazole (5 μM) for CYP2C9, ticlopidine (10 μM) for 2C19, quinidine (2 μM) for CYP2D6, diethyldithiocarbamate (50 μM) for CYP2E1, and ketoconazole (1 μM) for CYP3A4. The inhibition of the formation of M1, M2-9, M3-1, M3-2, M5-2, M5-3, M5-4, and M5-5 is expressed as percentage of the control (absence of inhibitor) and represent the means ± S.D. of triplicate experiments. Statistical significance was assessed using one-tailed student's *t*-test and is indicated with ^***^p < 0.005.

General or selective inhibitors of CYP isoforms were used to identify the CYPs responsible for the formation of M1, M2-9, M5-2, M5-3, M5-4, and M5-5 in HLMs. ABT, a general CYP inhibitor, was found to effectively inhibit (by 90%) the formation of M2-9, M5-2, M5-3, M5-4, and M5-5 and to moderately reduce (by 57%) M1 formation (Figure 4), suggesting the involvement of CYPs in the oxidation of F18. Specifically, ketoconazole (CYP3A4-selective) exerted a significant inhibitory effect (>80%) on the formation of M2-9, M5-2, M5-3, M5-4, and M5-5 and a slight inhibitory effect (20–30%) on the formation of M1 (Figure 5), suggesting that CYP3A4 might be a major CYP isoform mediating the oxidative metabolism of F18. Furthermore, quercetin (CYP2C8-selective) decreased the formation of M2-9, M5-2, and M5-3 by 10–30%, revealing that CYP2C8 also exhibits minor activity in the oxidative metabolism of F18. In contrast, only marginal inhibition (<10%) was observed with other inhibitors.

**Figure 6:**
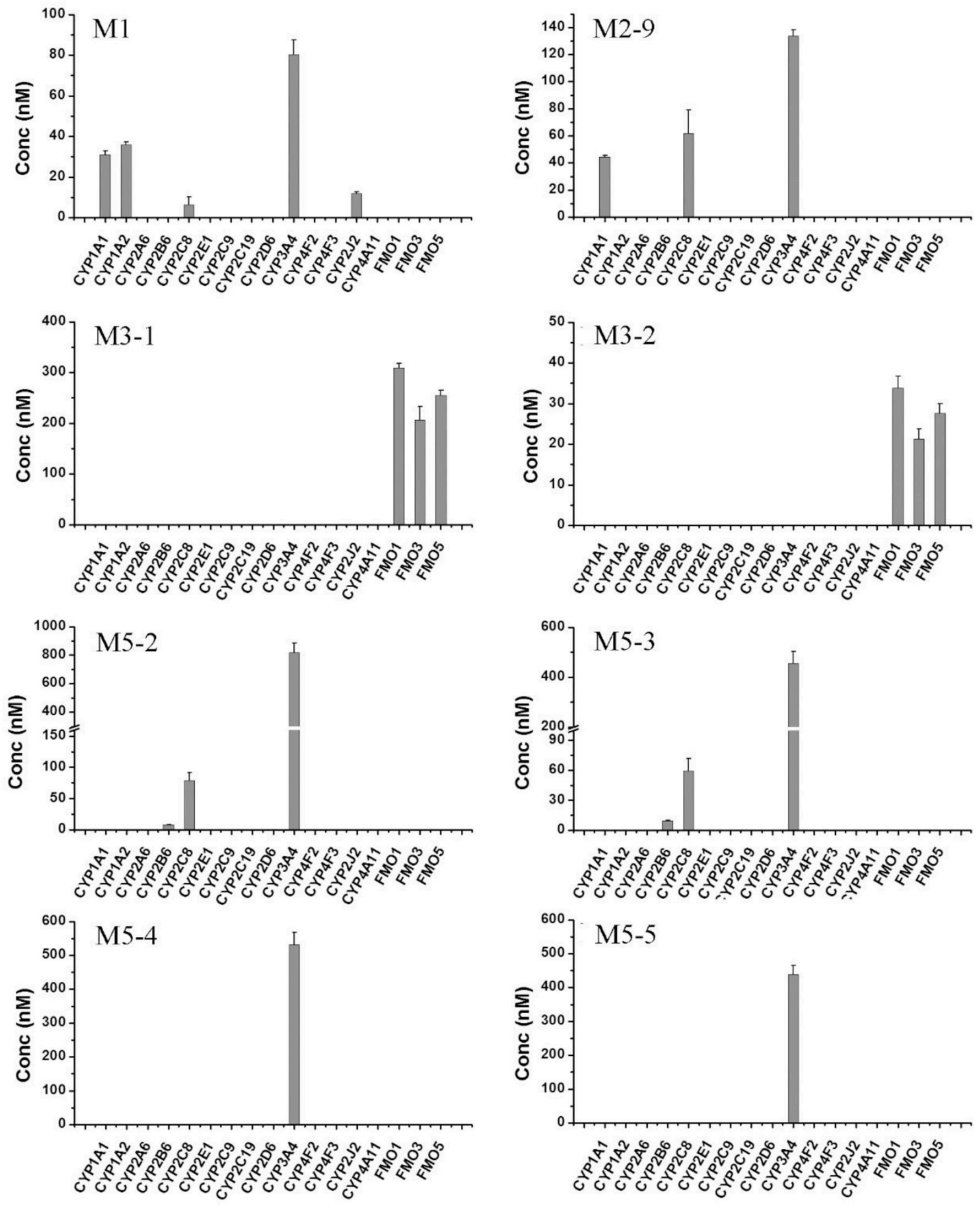
Formation of F18 metabolites in human recombinant CYP450s and FMOs. F18 (10 μM) was incubated individually with cDNA-expressed human CYP450 isozymes (50 pmol/mL CYP1A1, 1A2, 2A6, 2B6, 2C8, 2C9, 2C19, 2D6, 2E1, 2J2, 3A4, 4F2,4F3, or 4A11) or cDNA-expressed FMO enzymes (50 pmol/mL FMO1, FMO3, or FMO5) for 1 h. Each data represents the mean ± SD from three independent experiments.

To further confirm the roles of CYPs in F18 metabolism, the catalytic activities of 14 individual cDNA-expressed human CYP isoforms in F18 biotransformation were evaluated. As shown in Figure 6, the recombinant CYP3A4 showed the highest metabolic activity for the formation of M1, M2-9, M5-2, M5-3, M5-4, and M5-5, whereas the recombinant CYP2C8 was involved in the formation of M1, M2-9, M5-2, and M5-3. These results further confirmed the roles of CYP3A4 and CYP2C8 in the metabolism of F18. Moreover, recombinant CYP1A1, CYP1A2, and CYP2B6 might also participate in F18 metabolism to a certain extent, as evidenced by the generation of detectable amounts of one or two metabolites.

The role of FMOs in the oxidative metabolism of F18 was also investigated. The metabolism of F18 was effectively inhibited by the thermal inactivation of FMOs, resulting in a 64% reduction in M1 formation. Thus, FMOs might also play a role in F18 metabolism (Figure 4). However, no oxidative metabolites were observed in the incubations with recombinant FMO1, FMO3, and FMO5 (Figure 6).

#### Identification of the enzymes that catalyze the reduction of F18

F18 can be metabolized to M3-1 and M3-2 by the reduction of the carbonyl group in both HLMs and HLcy. The carbonyl reduction of ketone moieties is typically catalyzed by carbonyl reductases, such as ADH, AKRs, and SDRs. Therefore, the carbonyl reductase responsible for catalyzing the reduction reaction in F18 metabolism in HLMs and cytosol was investigated using chemical inhibitors (Table 4). In HLMs, the formation of M3-1 and M3-2 was slightly or moderately inhibited by 18β-glycyrrhetinic acid (an inhibitor of 11β-HSD) but not by 2-CEE (a CYP reductase inhibitor).

**Table 4:** Percentage of inhibition of the formation of M3-1 and M3-2 by chemical inhibitors in HLM and HLcy.

**Chemical inhibitor**	**Redutases**	**M3-1**		**M3-2**	
		**L**	**H**	**L**	**H**
HLM					
18β-Glycyrrhetinic	11β-HSD	1.2	13.7	51.4	67.5
2-CEE	NADPH-CYP450 reductase	N.I.	N.I.	N.I.	N.I.
**HLcy**					
Phenobarbital	AKR1A1,1B1	N.I.	N.I.	N.I.	N.I.
Flufenamic acid	AKR1C1,1C2,1C3,1C4	82.4	87.9	88.5	94.0
Chenodeoxycholic	AKR1C2	50.8	69.3	62.3	81.2
Medroxyprogesterone	AKR1C1,1C2,1C4	24.3	24.9	35.8	38.8
Quercetin	SDRs	41.5	80.4	54.6	87.0
Menadione	SDRs	7.7	45.9	20.1	58.4
Dicumarol (NADPH)	NQO1	52.3	70.6	67.7	82.2
Dicumarol (NADH)	NQO1	16.0	51.5	6.7	49.6
4-methylpyrazole (NADPH)	ADH	N.I.	N.I.	N.I.	N.I.
4-methylpyrazole (NADH)	ADH	N.I.	N.I.	N.I.	N.I.

*N.I., No inhibition*.

*L, Low concentration of inhibitor; H, high concentration of inhibitor*.

*HLM, human liver microsomes; HLcy, human liver microsomes; 11β-HSD, 11β-hydroxysteroid dehydrogenase; AKR, aldo-keto reductase; SDRs, short-chain dehydrogenases/reductases; NQO1, quinone oxidoreductase 1; ADH, alcohol dehydrogenase*.

In the cytosolic fraction, the formation of M3-1 and M3-2 was effectively inhibited (up to 94%) by flufenamic acid (a potent inhibitor of AKRs), chenodeoxycholic acid (a selective inhibitor of AKR1C2), and quercetin (an SDR inhibitor). The reduction activity was also moderately inhibited by medroxyprogesterone (a potent inhibitor of AKR1C1, 1C2, and 1C4) and menadione (an SDR inhibitor) in a concentration-dependent manner. In contrast, the formation of M3-1 and M3-2 was not affected by 10–50 μM phenobarbital (an inhibitor of AKR1A1 and AKR1B1). In contrast, dicumarol (an NQO1 inhibitor) but not 4-methylpyrazole (an ADH inhibitor) significantly inhibited the production of M3-1 and M3-2 in the presence of NADPH and NADH.

The roles of CYPs and FMOs in the reductive metabolism of F18 were also investigated (Figures 4–6). As expected, all CYP inhibitors used did not affect the formation of M3-1 and M3-2, and no experimental human cDNA-expressed CYP enzymes were found to catalyze the formation of M3-1 and M3-2. However, the formation of M3-1 was significantly inhibited by thermal inactivation of FMOs. Furthermore, both M3-1 and M3-2 were detected in the incubations with recombinant FMO1, FMO3, and FMO5.

### Pharmacokinetics of F18 in rats

**Figure 7:**
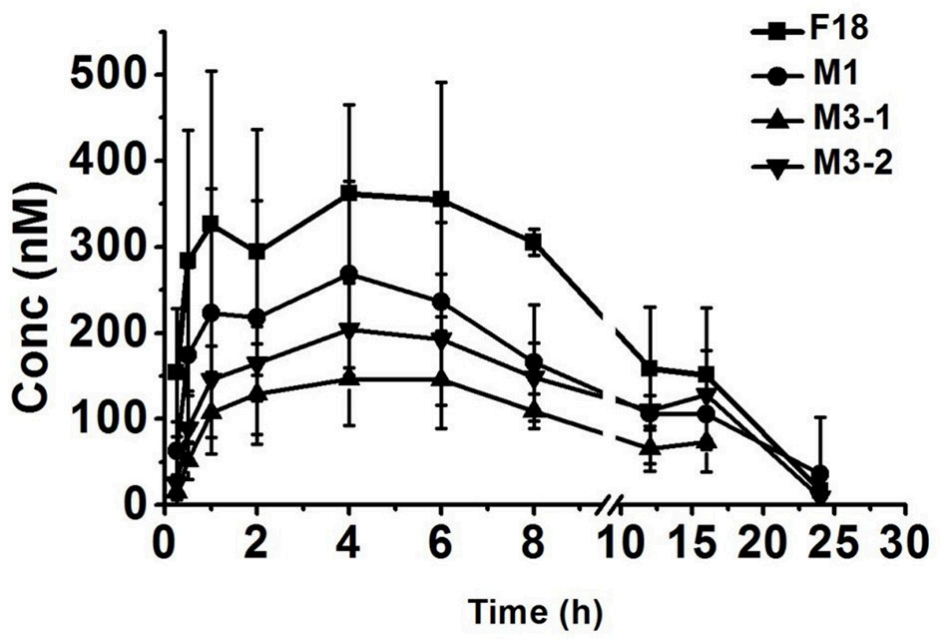
Mean blood concentration-time profiles of F18, M1, M3-1, and M3-2 in rats after oral administration of F18 at dose of 50 mg/kg (*n* = 5). The data are presented as the means ± SD. Conc., Concentration.

To determine the safety and efficacy profiles of F18, the blood exposures of the parent drug and metabolites were determined after the oral administration of F18 at 50 mg/kg. The oxidative dechlorination metabolites M1 and carbonyl reductive metabolites M3-1 and M3-2 were detected in high concentrations in rat blood, whereas the concentrations of the other metabolites were near or under the limit of detection. The blood concentration-time curves of F18, M1, M3-1, and M3-2 are shown in Figure 7, and non-compartmental pharmacokinetic parameters are listed in Table 5. The peak concentrations of F18, M1, M3-1, and M3-2 occurred at 3.3 to 4.0 h postdose and showed mean values of 443, 274, 165, and 219 nM, respectively, and the mean residence times (MRTs) were in the range of 8.9–13.2 h. The average area under the concentration-time curve (AUC_0−∞_) values of F18, M1, M3-1, and M3-2 were 5,166, 3,290, 1,965, and 3,353 h^*^nM, respectively. Therefore, the systemic exposures of M1, M3-1, and M3-2 were approximately 64, 38, and 65% of that of the parent drug, respectively.

**Table 5:** Pharmacokinetic parameters of F18 and its metabolites in rats after oral administration of F18 at 50 mg/kg.

**Parameter**	**F18**	**M1**	**M3-1**	**M3-2**
C_max_ (nM)	443 ± 107	274 ± 111	165 ± 50.9	219 ± 51.2
T_max_ (h)	3.3 ± 2.2	4.0 ± 1.6	4.0 ± 2.3	4.0 ± 2.3
AUC_0−t_ (h^*^nM)	*4, 627* ± *1, 454*	*3, 194* ± *1, 379*	*1, 935* ± 612	*2, 783* ± 747
AUC_0−∞_ (h^*^nM)	*5, 166* ± *1, 556*	*3, 290* ± *1, 347*	*1, 965* ± 615	*3, 353* ± 857
T_1/2_ (h)	5.3 ± 2.8	4.8 ± 2.1	3.4 ± 0.64	3.0 ± 0.4
MRT_0−t_ (h)	7.8 ± 1.0	8.4 ± 0.5	8.6 ± 0.6	8.8 ± 1.2
MRT_0−∞_ (h)	10.0 ± 2.4	9.4 ± 1.5	8.9 ± 0.7	13.2 ± 7.2

*Data are expressed as mean ± SD (n = 5)*.

### Anti-HIV-1 activities of F18 and its metabolites *In vitro*

The inhibition of HIV-1 replication against VSV-G/HIV-1 infections by F18 and its major metabolites was evaluated though a pseudovirus-based assay. The inhibitory activities of M2-9 (IC_50_: 0.11 μM) and M3-1/M3-2 (IC_50_: 0.034/0.051 μM) were more potent than that of F18 (IC_50_: 0.19 μM). In contrast, metabolites with hydroxylation at the 4-propyl moiety and oxidative dechlorination shown less potent activity than F18.

## Discussion

F18, a derivative of calanolide A, is an attractive antiretroviral candidate for the treatment of HIV. Combination therapy is the most effective strategy for HIV-infected patients; thus, gaining a comprehensive understanding of the metabolic pathways involved in the clearance of F18 is essential.

### Identification of F18 metabolites *In vitro* and *In vivo*

Metabolite profiling is a useful tool for predicting the pharmacokinetic properties of drug candidates and assessing the possibility of drug-drug interactions (DDIs). Detecting and characterizing reactive metabolites in both experimental animals and humans are highly important initial steps in evaluating the underlying risk and can, in turn, guide the rational design of compounds that do not lead to the formation of reactive metabolites (Evans and Baillie, 2005). Furthermore, an early awareness of the pharmacological activities of major metabolites could facilitate the design of appropriate studies to better investigate the implications of such metabolites, particularly if they have biological activities similar to that of the parent drug (Mutlib et al., 2011).

In the present study, the metabolism of F18 was comprehensively investigated using liver microsomes from different species and human liver cytosol *in vitro*. F18 was observed to be extensively metabolized in liver microsomes: 23 metabolites were detected, and all of them were oxidative metabolites, with the exception M3-1 and M3-2, which were carbonyl reduction metabolites. As expected, the carbonyl reduction metabolites were also observed in the incubation in HLcy. Furthermore, M1, M3-1, and M3-2 are stable metabolites *in vivo* and were also observed in blood with a high exposure.

The multiple metabolic sites contributing to F18 metabolism were mainly identified at three locations: 10-chloromethyl (M1), 4-propyl chain (M2-9, M5-2, M5-3, M5-4, and M5-5), and 12-carbonyl (M3-1 and M3-2). Because of their low relative amounts compared with that of the major metabolites, the metabolites with ambiguous structures are not discussed in this manuscript. In the case of 10-chloromethyl, the oxidative dechlorination metabolite M1 exhibited inhibitory activity against HIV-1, although its potency was only one fifth that of the parent compound. Because it is relatively stable [its blood concentration reaches a high steady state *in vivo* (65% that of the parent compound)], M1 is believed to be the major metabolite that contributes to the pharmacological effects of F18. The secondary metabolic pathway of F18 involves oxidation of the 4-propyl chain. Several hydroxylated (M5-2, M5-3, M5-4, and M5-5) and dehydrogenated (M2-9) metabolites involving the 4-propyl chain were identified. Hydroxylation of the 4-propyl group resulted in lower anti-HIV activities, whereas dehydrogenation metabolites showed activities similar to that of the parent compound. These results confirmed that the hydrophobic 4-propyl chain is necessary for the inhibitory activity against HIV-1 *in vitro* (Xue et al., 2010). However, the exposures of hydroxylated and dehydrogenated metabolites were relatively low *in vivo*. Interestingly, because calanolide A also contains a 4-propyl group, oxidation of the 4-propyl chain might occur in other calanolide A analogs. M3-1 and M3-2 were identified as the major metabolites in both HLMs and HLcy, indicating that reduction of the 12-carbonyl group was the most important metabolic pathway of F18. In general, the reduction of xenobiotics is considered an inactivation and detoxification reaction because the resulting alcohol is easier to conjugate and eliminate (Maser, 2004). However, in the present study, the reduction metabolites of F18 exhibited favorable inhibitory activities against HIV-1. The inhibitory activities of M3-1 and M3-2 against HIV-1 were 2–3 folds greater than that of the parent compound. Additionally, for chemists, the 12-carbonyl group is an important modification site of calanolide A; therefore, the reductive metabolic pathway should be seriously considered when developing analogs of 12-oxo-calanolide A (Sekino et al., 2004; Ma et al., 2008). Furthermore, the better inhibitory activities and high exposures of M3-1 and M3-2 might contribute to the pharmacological effects of F18.

To obtain a sufficiently clear response in the detected metabolites, we selected 50 μM as the incubated concentration. The metabolic profiles for different incubated concentration were also compared. The major metabolite profiles obtained with low and high concentrations of F18 in the incubation did not show significant differences, whereas only a slight difference in the metabolic site of dehydrogenation was observed. Our previous study showed that F18 exhibited potent inhibitory effects on CYP2C9, CYP2C19, and CYP1A2 and activation-inducing effect on CYP3A4 (unpublished data). The slight metabolism difference between low and high concentrations of F18 might be due to the effects of F18 on drug metabolizing enzymes.

### Drug metabolizing enzymes responsible for F18 metabolism

**Figure 8:**
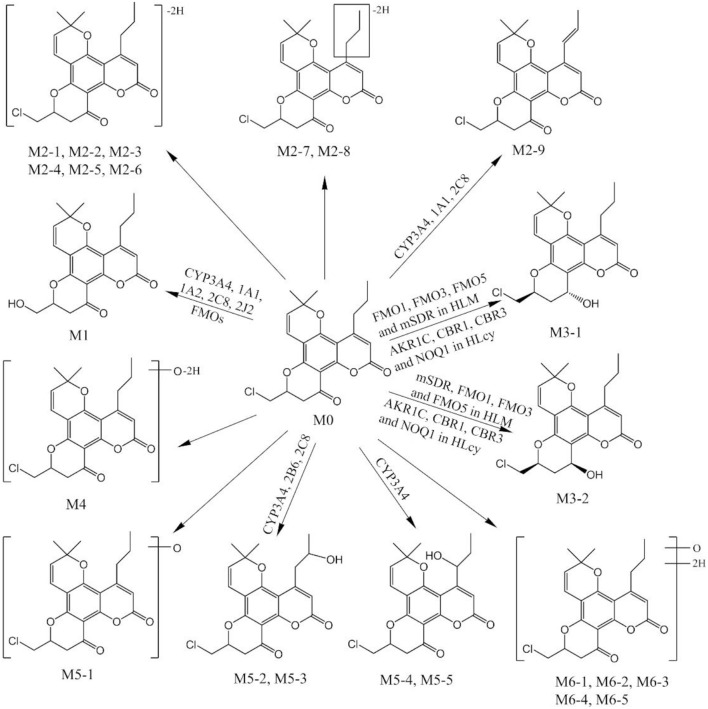
Proposed metabolic pathway of F18 *in vitro*.

The second part of this investigation focused on identifying the enzymes involved in F18 metabolism. Figure 8 summarizes the *in vitro* metabolic pathways of F18, including the drug metabolizing enzymes putatively related to the formation of the major metabolites. Due to their relative low amount, the enzymes responsible for the formation of the minor metabolites were not clarified in the present study.

CYPs, which constitute a heme-containing superfamily, are responsible for the bulk of the oxidative metabolism of known drugs in humans (Wienkers and Heath, 2005). In this work, we first identified the enzymes responsible for the oxidative metabolism of F18. As expected, the use of chemical inhibitors and recombinant enzymes revealed that multiple CYPs, particularly the CYP3A4 isoform, which catalyzes reactions at diverse metabolic sites, are involved in the oxidative metabolism of F18. Indeed, human CYP3A4 has a flexible substrate-binding pocket capable of accommodating a broad range of substrates of varying sizes (Ekroos and Sjogren, 2006). Because CYP3A4 is present not only in the liver but also in the small intestine where it functions as a barrier against xenobiotics, both liver and intestinal first-pass metabolism might affect the bioavailability of F18 after oral administration. Moreover, in the treatment for HIV, F18 might have to be taken concurrently with other antiretroviral drugs that are substrates, inhibitors and inducers of CYP3A4. For instance, etravirine, a second-generation NNRTI, has been found to increase the abundance of CYPP3A4 mRNA and to induce the metabolism of other CYP3A4 substrates (Yanakakis and Bumpus, 2012). Therefore, the potential DDI mediated by CYP3A4 should be investigated further.

In addition to CYP3A4, CYP2C8 was also found to be responsible for the formation of M1, M2-9, M5-2, and M5-3. CYP2C8, a major human hepatic CYP, constitutes approximately 7% of the total microsomal CYP content in the liver. Although the polymorphisms of CYP2C8 are closely related to individually variant responses to drugs, they might not affect the metabolism of F18 due to the low contribution of CYP2C8 (Jiang et al., 2011).

FMOs constitute one of the most important non-CYP enzymes involved in the oxidative metabolism of xenobiotics. In general, FMOs typically catalyze the addition of an oxygen atom to substrates that contain a nucleophilic heteroatom, such as nitrogen, sulfur, phosphorus or selenium (Fan et al., 2016). However, due to the lack of effective and specific inhibitors, the contributions of FMOs cannot be distinguished from those of CYPs. In contrast to CYPs, FMOs are not readily irreversibly inhibited by different chemicals (Kedderis and Rickert, 1985). Instead, because FMOs are thermally labile in the absence of NADPH, thermal inactivation studies are often performed to evaluate the involvement of FMOs in drug metabolism (Fan et al., 2016). After thermal inactivation, the formation of M1 was effectively inhibited, indicating the contribution of FMOs to the production of M1. The reliability of these conclusions is supported by the following results: (1) The metabolism of midazolam, a CYP3A4 substrate, was unchanged. (2) The metabolism of benzydamine, an FMO substrate, was effectively inhibited in the incubation system. FMOs have been reported to catalyze oxidative defluorination (Fan et al., 2016). Our results might provide a new scenario in which FMOs catalyze oxidative dehalogenation. However, M1 was not detected in the incubations with recombinant FMO1, FMO3, and FMO5. Humans possess five functional FMOs, designated FMO1-FMO5. FMO1 has the broadest substrate range, followed by FMO3, whereas FMO2 and FMO5 have more restricted ranges of substrates, and almost nothing is known regarding the substrate range of FMO4 (Phillips and Shephard, 2017). Therefore, the FMO isoforms responsible for the formation of M1 require further study. Thus, multiple CYPs (CYP1A1, CYP1A2, CYP2B6, CYP2J2, and CYP3A4) and FMOs are responsible for the oxidative metabolism of F18.

Ketones can only be reduced by carbonyl reducing enzymes, generating their respective secondary hydroxyl metabolites (Rosemond and Walsh, 2004). 11β-HSD and microsomal CYP reductase are two well-documented enzymes responsible for microsomal carbonyl reduction in humans (Skarydova and Wsol, 2012). Interestingly, two carbonyl reduction metabolites, identified as M3-1 and M3-2, were observed in the HLM incubations in our study. 18β-Glycyrrhetinic acid, an 11β-HSD inhibitor (Stewart et al., 1987; Maser et al., 1996, 2003), effectively inhibited the formation of M3-2 but had no significant effect on the formation of M3-1. However, 2-CEE, a CYP reductase inhibitor (Gray et al., 2010), was ineffective. These results indicate that 11β-HSD is the major enzyme involved in the carbonyl reduction metabolism of F18. However, no enzyme has been found to be responsible for the formation of M3-1. Interestingly, heat inactivation of microsomal FMOs effectively decreased the formation of M3-1, suggesting that FMOs might be involved in its formation. Furthermore, both M3-1 and M3-2 were detected in the incubations with recombinant FMO1, FMO3, and FMO5. As described above, FMO superfamily members mainly catalyze heteroatom oxidation, particularly that of nucleophilic atoms (Fan et al., 2016). To the best of our knowledge, the carbonyl reduction reactions catalyzed by FMOs have not been described previously. A recent study reported that human FMO5 could catalyze the oxidation of a wide range of carbonyl compounds via the insertion of an oxygen atom into a carbon-carbon bond adjacent to the carbonyl group to form an ester; however, this enzyme is not involved in reduction (Phillips and Shephard, 2017). The mechanism through which FMOs catalyze carbonyl reductions requires further study. In summary, 11β-HSD and FMOs are currently considered major microsomal enzymes involved in F18 carbonyl reduction.

Our data show that the carbonyl reduction of F18 occurs not only in microsomes but also in the liver cytosol in the presence of NADPH or NADH. Because the liver cytosol is rich in various enzymes responsible for carbonyl reduction, the enzymes involved in the carbonyl reduction of F18 in the liver cytosol were also identified. The enzymes that catalyze the reduction of aldehyde and ketone moieties in the liver cytosol include AKRs, ADHs, SDRs and NQO1 (Rosemond and Walsh, 2004; Skarydova and Wsol, 2012). These oxidoreductases function in biological processes are widely, such as steroid and prostaglandin metabolism, antioxidant effects, stress response, and carcinogenesis (Maser, 1995; Oppermann et al., 2001; Chang et al., 2003; Nam et al., 2014). Among these enzymes, AKRs and SDRs are NADPH-dependent oxidoreductases. The AKR1 family, which contains members of the AKR1A1 (aldehyde reductase), AKR1B1 (aldose reductase), AKR1C (HSDs) and AKR1D (steroid 5β-reductases) subfamilies, is by far the largest and most prevalent family involved in carbonyl reduction (Barski et al., 2008). In the present study, phenobarbital, an inhibitor of AKR1A1 and AKR1B1 (Rosemond and Walsh, 2004; Tong et al., 2010), had no inhibitory effect on the formation of M3-1 and M3-2. In contrast, all of the experimental inhibitors of the AKR1C subfamily, including flufenamic acid, chenodeoxycholic acid and medroxyprogesterone (Atalla et al., 2000; Rosemond and Walsh, 2004; Steckelbroeck et al., 2006), exhibited significant inhibitory effects on the formation of M3-1 and M3-2, indicating that the AKR1C subfamily plays an important role in F18 metabolism. The human AKR1C subfamily includes four isoforms—AKR1C1, AKR1C2, AKR1C3, and AKR1C4—that share over 86% homology (Barski et al., 2008). Due to the lack of a specific inhibitor, the isoforms' contributions to carbonyl reduction should be further investigated.

Several members of the SDR superfamily, including cytosolic CBR1 and CBR3, are known to reduce xenobiotic carbonyl compounds. Among these enzymes, CBR1 has a much broader substrate specificity and higher catalytic efficiency than CBR3 (Hoffmann and Maser, 2007). Quercetin, a CBR1 inhibitor (Atalla et al., 2000; Rosemond and Walsh, 2004), significantly inhibited the formation of M3-1 and M3-2, indicating that CBR1 is involved in F18 metabolism. Menadione, another CBR inhibitor that reportedly does not affect the activity of CBR1, can also inhibit the carbonyl reduction of F18 (Lehr et al., 2015). Thus, CBR3 might also participate in the formation of M3-1 and M3-2.

Unlike the AKR and SDR superfamilies, NQO1 and ADHs are NADH- or NADPH-dependent oxidoreductases (Rosemond and Walsh, 2004). Because the carbonyl reduction of F18 occurred in the presence of both NADH and NADPH, the roles of NQO1 and ADH were evaluated under the above conditions. NQO1 was mainly responsible for the reduction of quinones, while ADH was involved in the reduction of aldehydes (Walsh et al., 2002; Cullen et al., 2003). In the present study, irrespective of whether NADH or NADPH was used as a cofactor, the formation of M3-1 and M3-2 was inhibited by a NQO1 inhibitor (dicumarol) while not by ADH inhibitor (4-methylpyrazole), indicating the role of NQO1 in the carbonyl reduction of F18 (Rosemond and Walsh, 2004). Altogether, multiple carbonyl reductases, including AKR1C, SDRs, and NOQ1, were found to be responsible for the formation of M3-1 and M3-2 in HLcy.

## Conclusion

In conclusion, F18 was found to be extensively metabolized in HLMs and HLcy, and 4-propyl chain, 10-chloromethyl oxidation and 12-carbonyl reduction are considered the major metabolic pathways of F18. The carbonyl reduction metabolites of F18 exhibit favorable inhibitory activities against HIV-1 and high exposure in rats after oral administration. Multiple drug metabolizing enzymes, including CYPs, FMOs and a variety of carbonyl reductases, are believed to be involved in F18 metabolism. Notably, the present results provide the first demonstration of the capability of FMOs for carbonyl reduction.

## Ethics statement

This study was carried out in accordance with the recommendations of guidelines for Animal Experimental Center, Animal Care and Welfare Committee, Institute of Materia Medica, CAMS and PUMC. The protocol was approved by the Animal Care and Welfare Committee, Institute of Materia Medica, CAMS PUMC.

## Author contributions

Participated in the research design: XW, LS, and YJ. Conducted the experiments: XW, QZ, JG, ZZ, MZ, and YY. Contributed new reagents or analytic tools: XW, JH, and BW. Performed the data analysis: XW, LS. Wrote or contributed to the writing of the manuscript: XW, LS, and YL.

### Conflict of interest statement

The authors declare that the research was conducted in the absence of any commercial or financial relationships that could be construed as a potential conflict of interest.

## Abbreviations

NMR: Nuclear magnetic resonance
RT: reverse transcriptase
NNRTI: nonnucleoside reverse transcriptase inhibitor
VSV-G: vesicular stomatitis virus glycoprotein
6-G-P: glucose-6-phosphate
6-G-P-DH: 6-G-P dehydrogenase
ABT: aminobenzotriazole
2-CEE: 2-chloroethyl ethyl sulfide
HPLC: high-performance liquid chromatography
CYPs: cytochrome P450s
FMOs: flavin-containing monooxygenases
HLMs: human liver microsomes
RLMs: rat liver microsomes
DLMs: dog liver microsomes
MLMs: monkey liver microsomes
HLcy: human liver cytosol
LC-MS/MS: liquid chromatography-tandem mass spectrometry
ROESY: Overhauser effect correlation spectroscopy
11β-HSD: 11β-hydroxysteroid dehydrogenase
AKRs: aldo-keto reductases
SDRs: short-chain dehydrogenases/reductases
NQO1: quinone oxidoreductase 1
ADHs: alcohol dehydrogenases
CBRs: carbonyl reductases.
