# Supplementary material for: Metabolism of F18, a Derivative of Calanolide A, in Human Liver Microsomes and Cytosol
Source: Front Pharmacol. 2017 Jul 19;8:479. doi: 10.3389/fphar.2017.00479 (PMC5515859; doi:10.3389/fphar.2017.00479)
Supplement: Supplementary file 1 [file DataSheet1.pdf]

Supplemental data

Supplemental Table S1

Table of *in vitro* selective substrates, inhibitors and Inducers for P450-mediated metabolism.

CYPs	Inhibitor[1]				Sbutrate		
	preferred		Accetable				
	inhibitor	Ki (uM)	inhibitor	Ki (uM)	substrate	Km (uM)	metabolite
1A2	furafylline[2]	0.6-0.73	α-naphthoflavone	0.01	Phenacetin	1.7-152	Acetaminophen
2A6	Tranylcypromine<br>methoxsalen[2]	0.02-0.2<br>0.01-0.2	Pilocarpine<br>tryptamine	4<br>1.7	coumarin	0.3-0.23	coumarin-7-hydroxylation
2B6			Sertraline<br>Phencyclidine<br>Clopidogrel<br>ticlopidine	3.2<br>10<br>0.5<br>0.2	bupropion	67-168	bupropion-hydroxylation
2C8	Quercetin<br>montelukast	1.1	Trimethoprim<br>pioglitazone	32<br>1.7	Taxol<br>amodiaquine	5.4-19<br>2.4	Taxol 6-hydroxylation<br>*N*-Des*ethylAmodiaquine*
2C9	sulfaphenazole	0.3	Fluconazole<br>fluoxetine	7<br>18-41	Diclofenac	3.4-52	4-Hydroxydiclofenac
2C19			Ticlopidine<br>nootkatone	1.2<br>0.5	S-mephenytoin	13-35	4-Hydroxymephenytoin
2D6	quinidine	0.027-0.4			dextromethorphan	0.44-8.5	Dextrophan
2E1			Diethyldithiocarbamate<br>Clomethiazole<br>diallyldisulfide	9.8-3.4<br>12<br>150	chlorzoxazone	39-157	6-Hydroxychlorzoxazone
3A4/5	Ketoconazole<br>itraconazole	0.0037-0.18<br>0.27,2.3	Troleandomycin<br>verapamil	17<br>10,24	Midazolam<br>testosterone	1-14	1-Hydroxymidazolam<br>6-hydroxytestosterone

Supplemental Figure S1

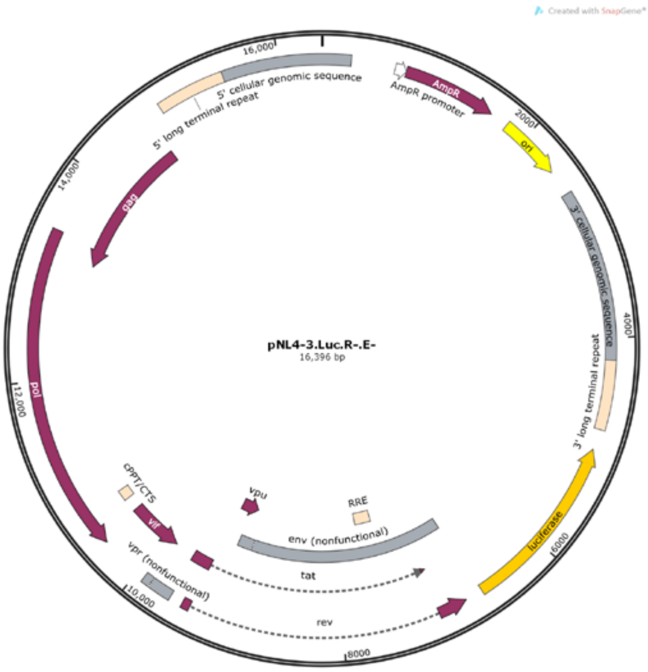

Supplemental Figure S1 Map of pNL4-3.Luc.R^-E^- plasmid was adopted from National Institute of Health AIDS Research and Reference Reagent Program Website. (https://www.aidsreagent.org/reagentdetail.cfm?t=cloning_vectors&id=66)

Supplemental Figure S2

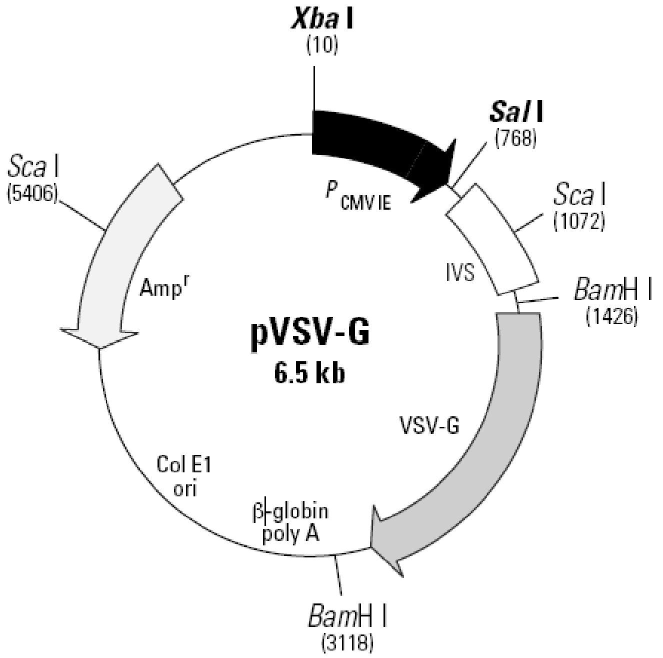

Supplemental Figure S2 Plasmid map of VSV-G

Supplemental Figure S3

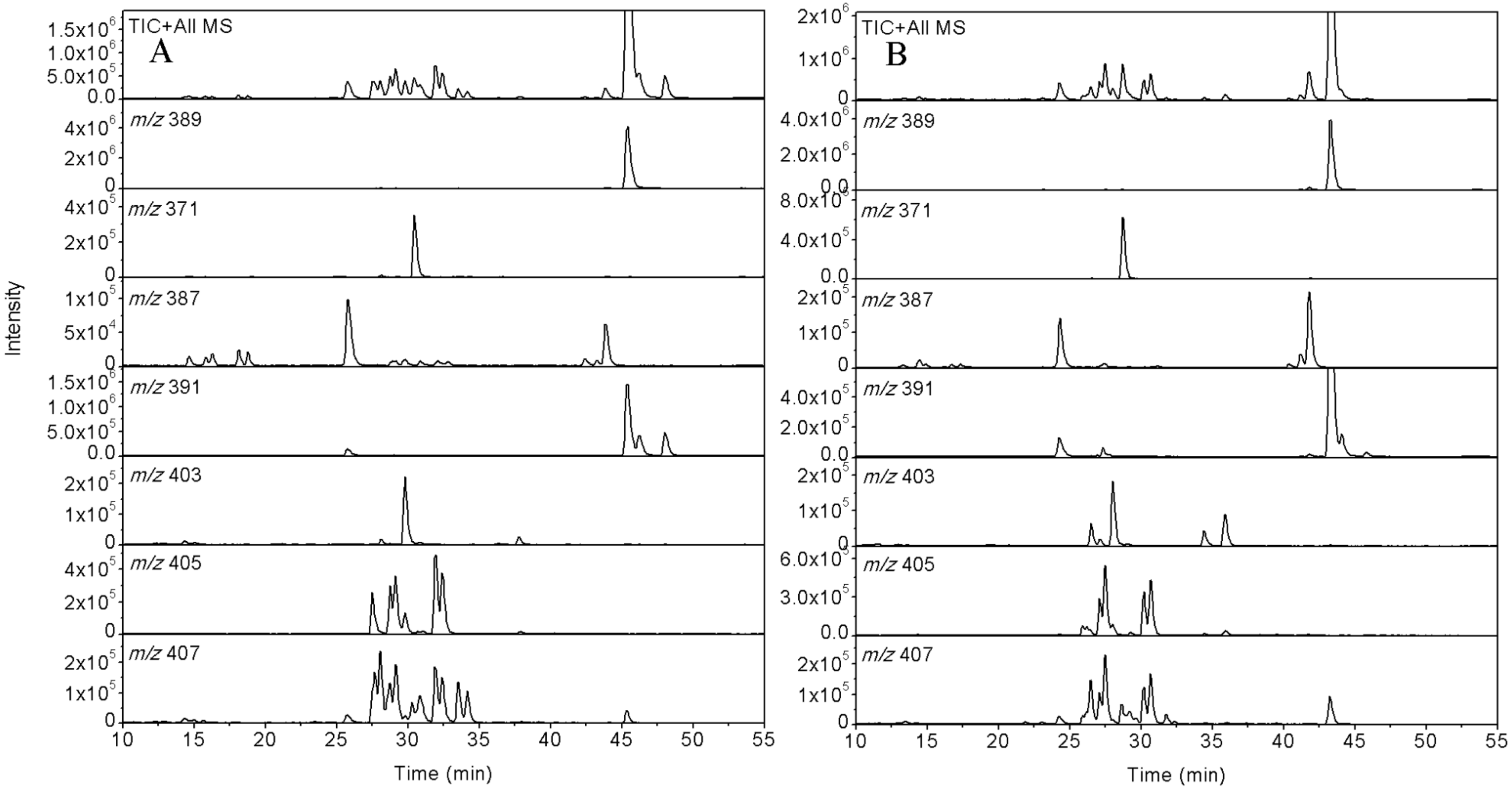

Supplemental Figure S3 (continued)

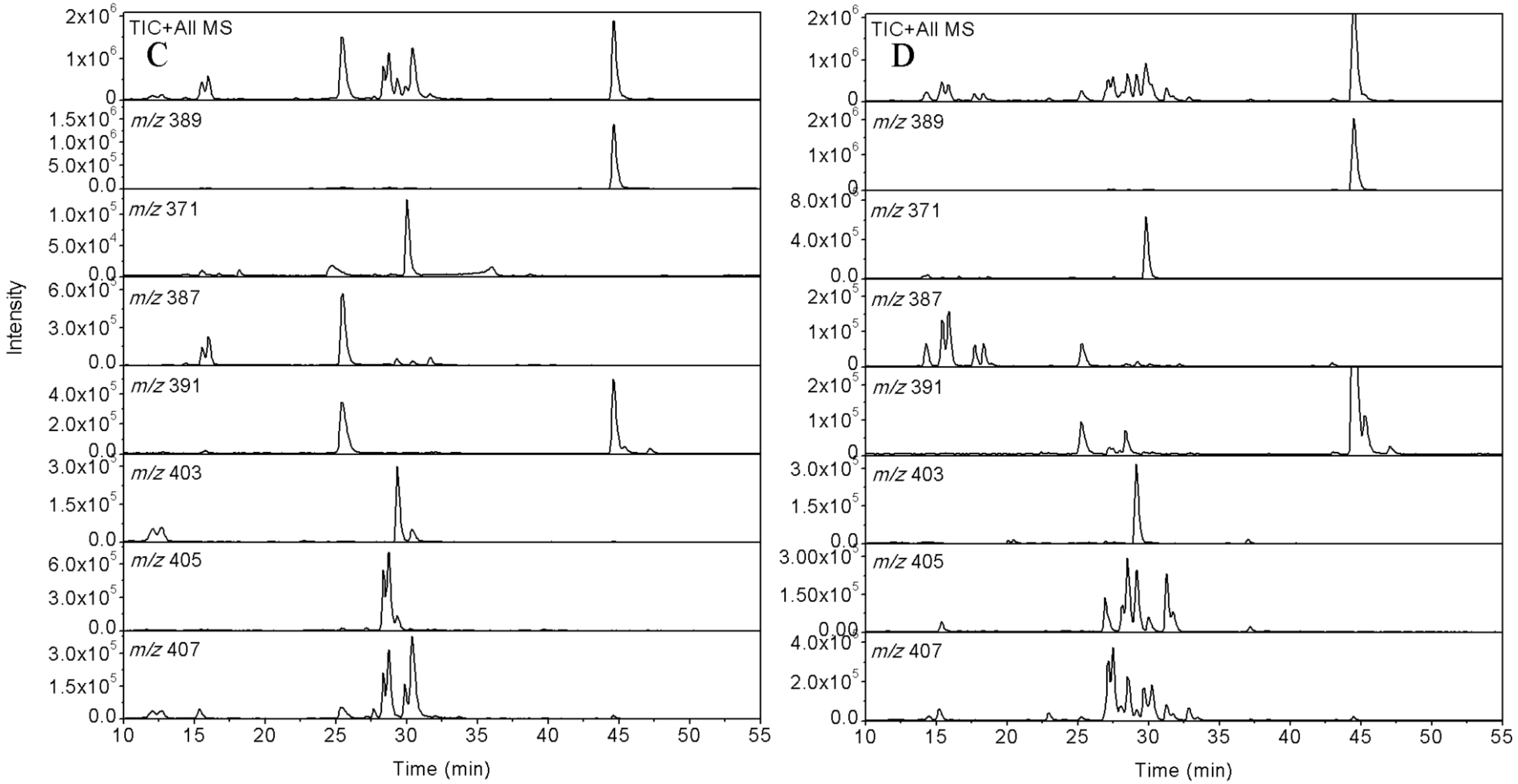

Supplemental Figure S3 Extracting ion $[M+H]^+$ chromatograms of F18 and its metabolites in HLM/RLM/DLM/MLM incubation with NADPH-

regenerating system

A: HLM; B: RLM; C: DLM; D: MLM.

Supplemental Figure S4

A

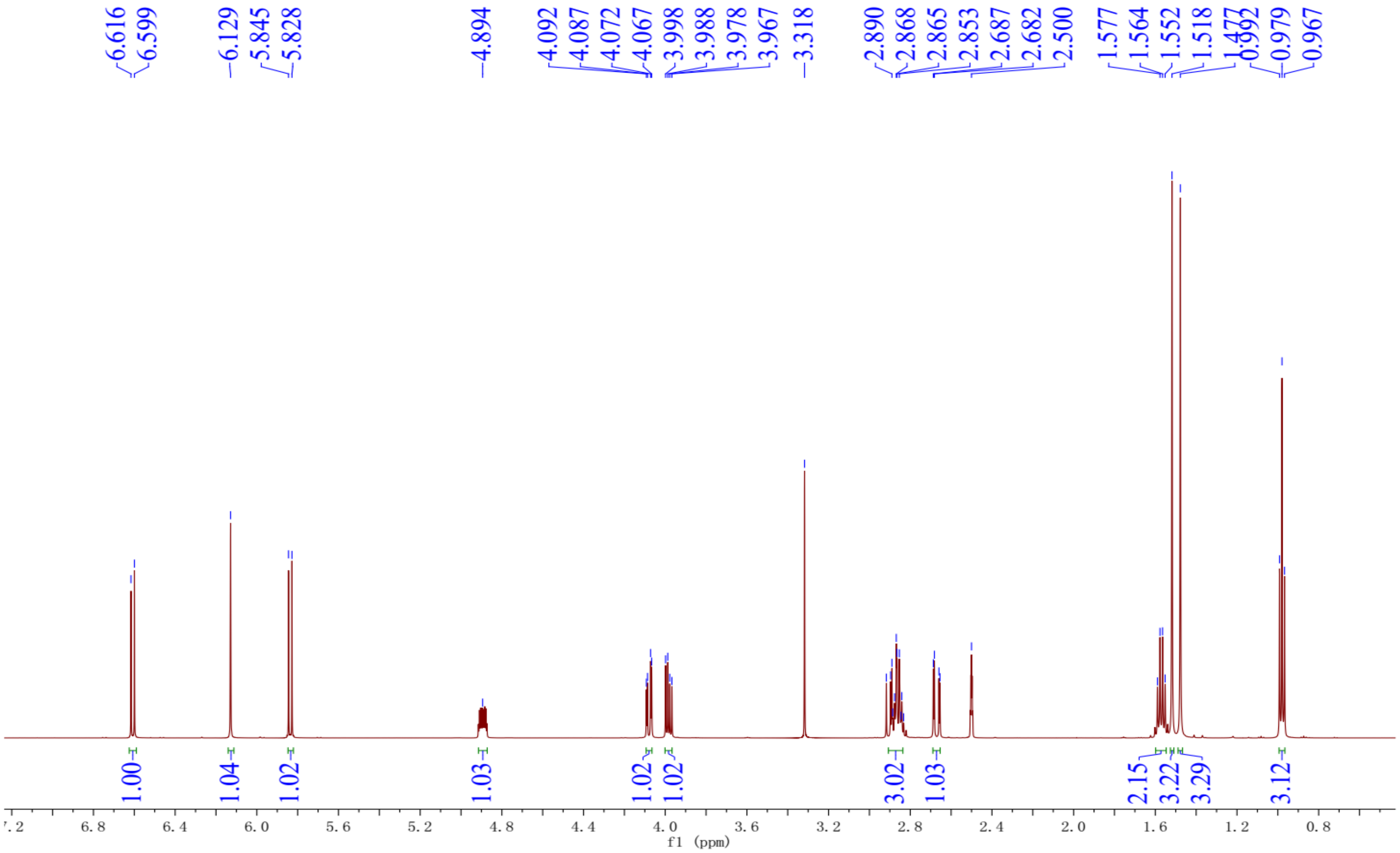

B

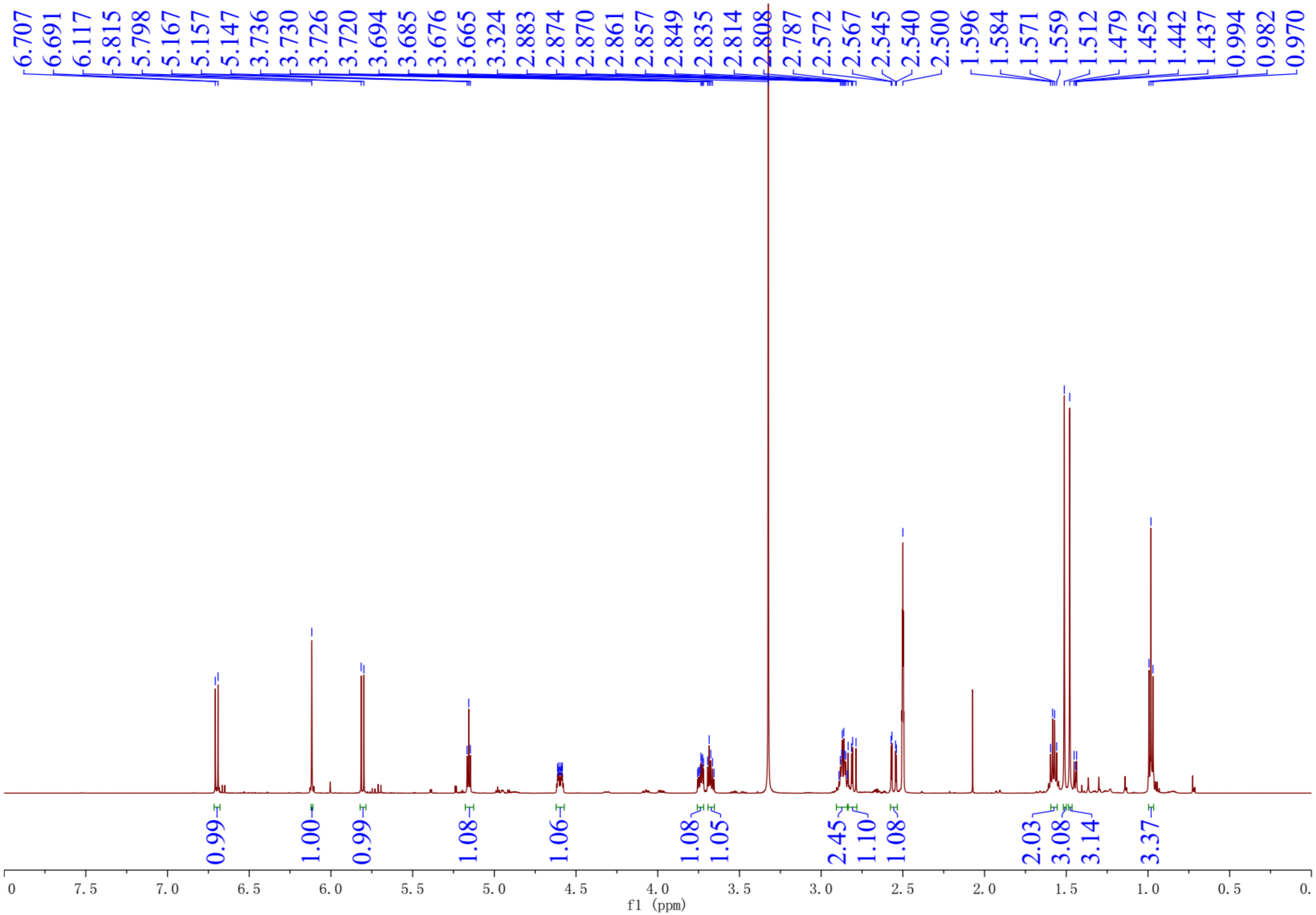
6.707
6.691
6.117
5.815
5.798
5.167
5.157
5.147
3.736
3.730
3.726
3.720
3.694
3.685
3.676
3.665
3.324
2.883
2.874
2.870
2.861
2.857
2.849
2.835
2.814
2.808
2.787
2.572
2.567
2.545
2.540
2.500
1.596
1.584
1.571
1.559
1.512
1.479
1.452
1.442
1.437
0.994
0.982
0.970
0.99
1.00
0.99
1.08
1.06
1.08
1.05
2.45
1.10
1.08
2.03
3.08
3.14
3.37
0
7.5
7.0
6.5
6.0
5.5
5.0
4.5
4.0
3.5
3.0
2.5
2.0
1.5
1.0
0.5
0.
f1 (ppm)

25

C

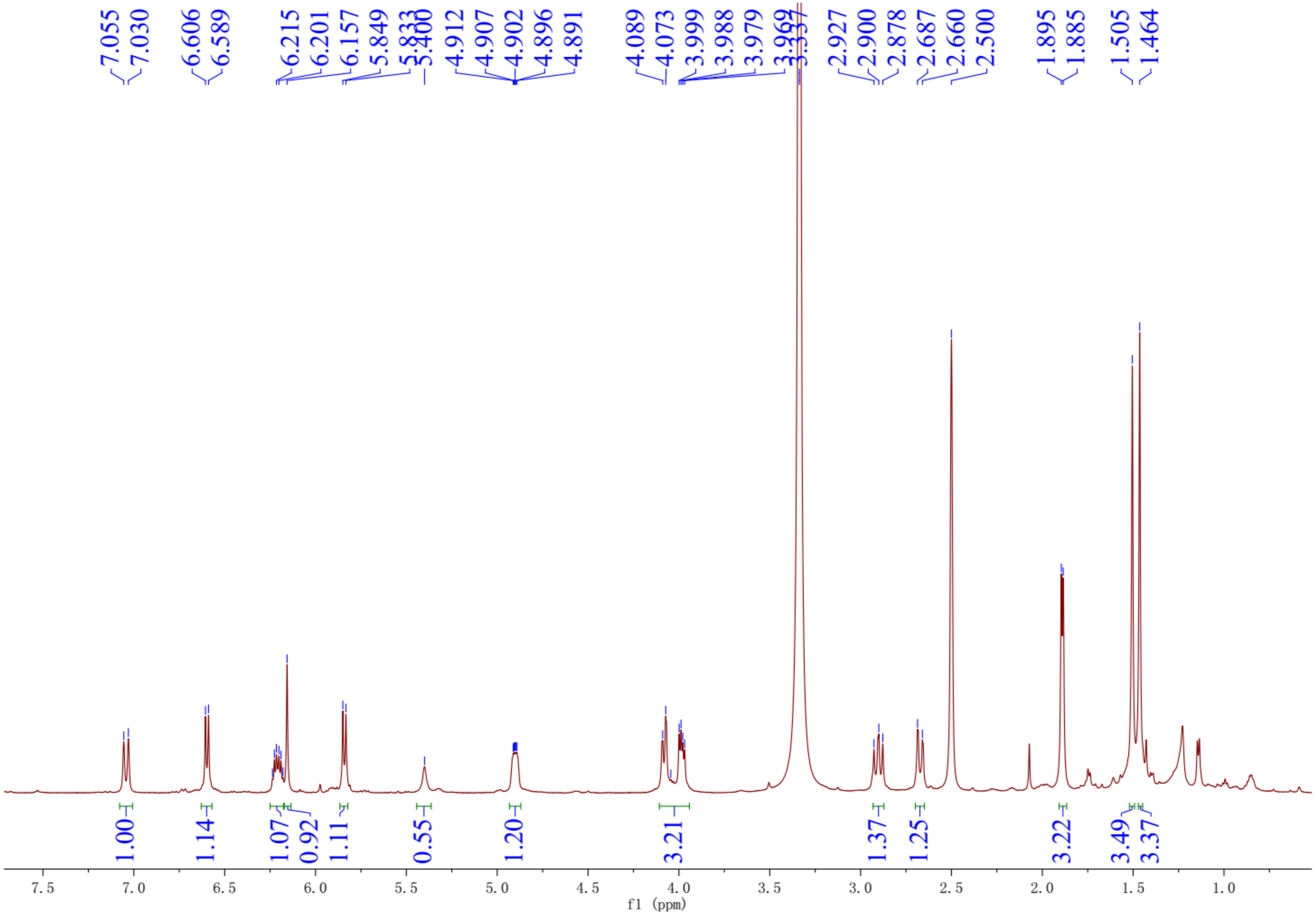
7.055
7.030
6.606
6.589
6.215
6.201
6.157
5.849
5.833
5.400
4.912
4.907
4.902
4.896
4.891
4.089
4.073
3.999
3.988
3.979
3.969
3.357
2.927
2.900
2.878
2.687
2.660
2.500
1.895
1.885
1.505
1.464
1.00
1.14
1.07
0.92
1.11
0.55
1.20
3.21
1.37
1.25
3.22
3.49
3.37
7.5
7.0
6.5
6.0
5.5
5.0
4.5
4.0
3.5
3.0
2.5
2.0
1.5
1.0
f1 (ppm)

26

D

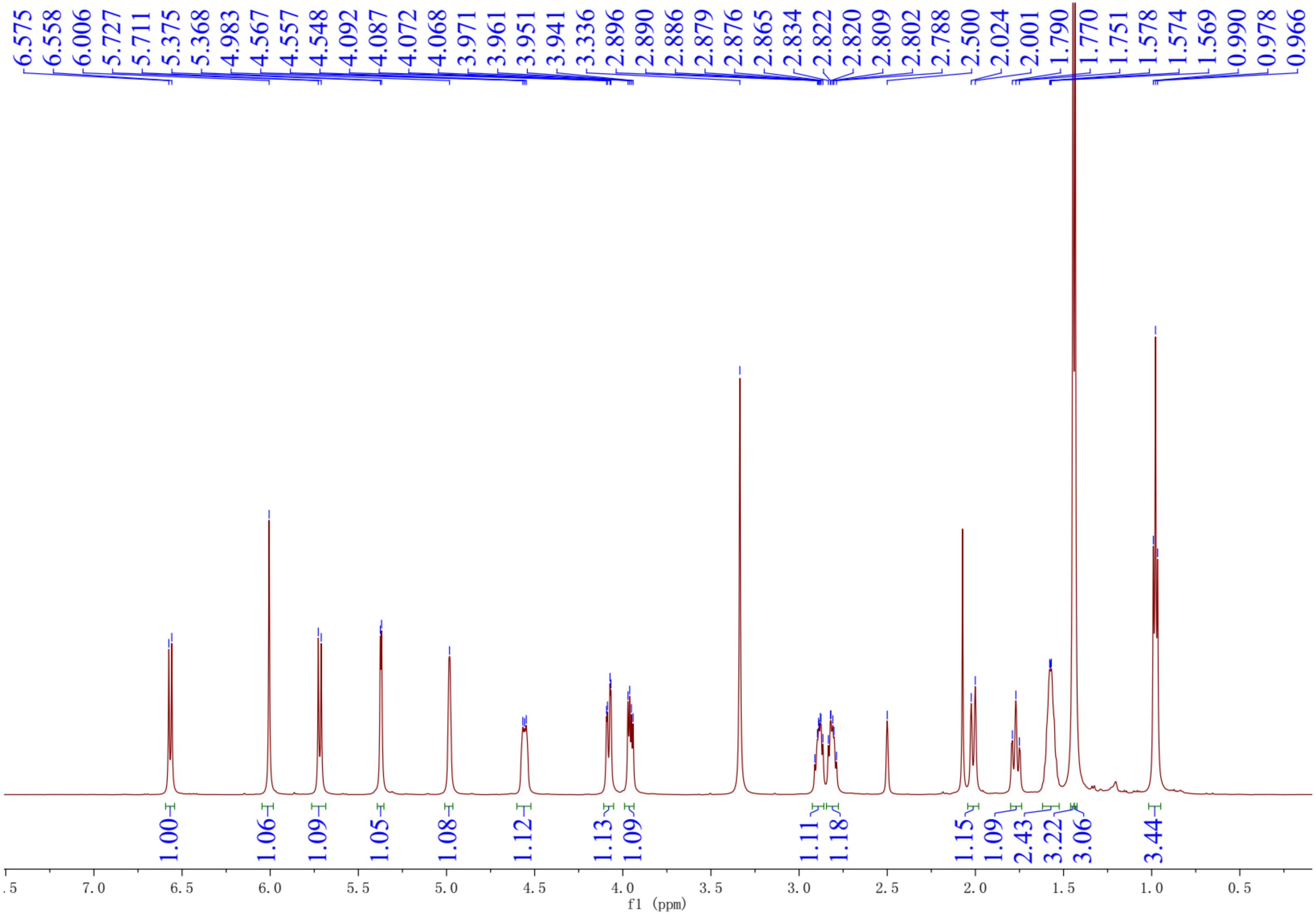

6.575
6.558
6.006
5.727
5.711
5.375
5.368
4.983
4.567
4.557
4.548
4.092
4.087
4.072
4.068
3.971
3.961
3.951
3.941
3.336
2.896
2.890
2.886
2.879
2.876
2.865
2.834
2.822
2.820
2.809
2.802
2.788
2.500
2.024
2.001
1.790
1.770
1.751
1.578
1.574
1.569
0.990
0.978
0.966
1.00
1.06
1.09
1.05
1.08
1.12
1.13
1.09
1.11
1.18
1.15
1.09
2.43
3.22
3.06
3.44
.5
7.0
6.5
6.0
5.5
5.0
4.5
4.0
3.5
3.0
2.5
2.0
1.5
1.0
0.5
f1 (ppm)

27

E

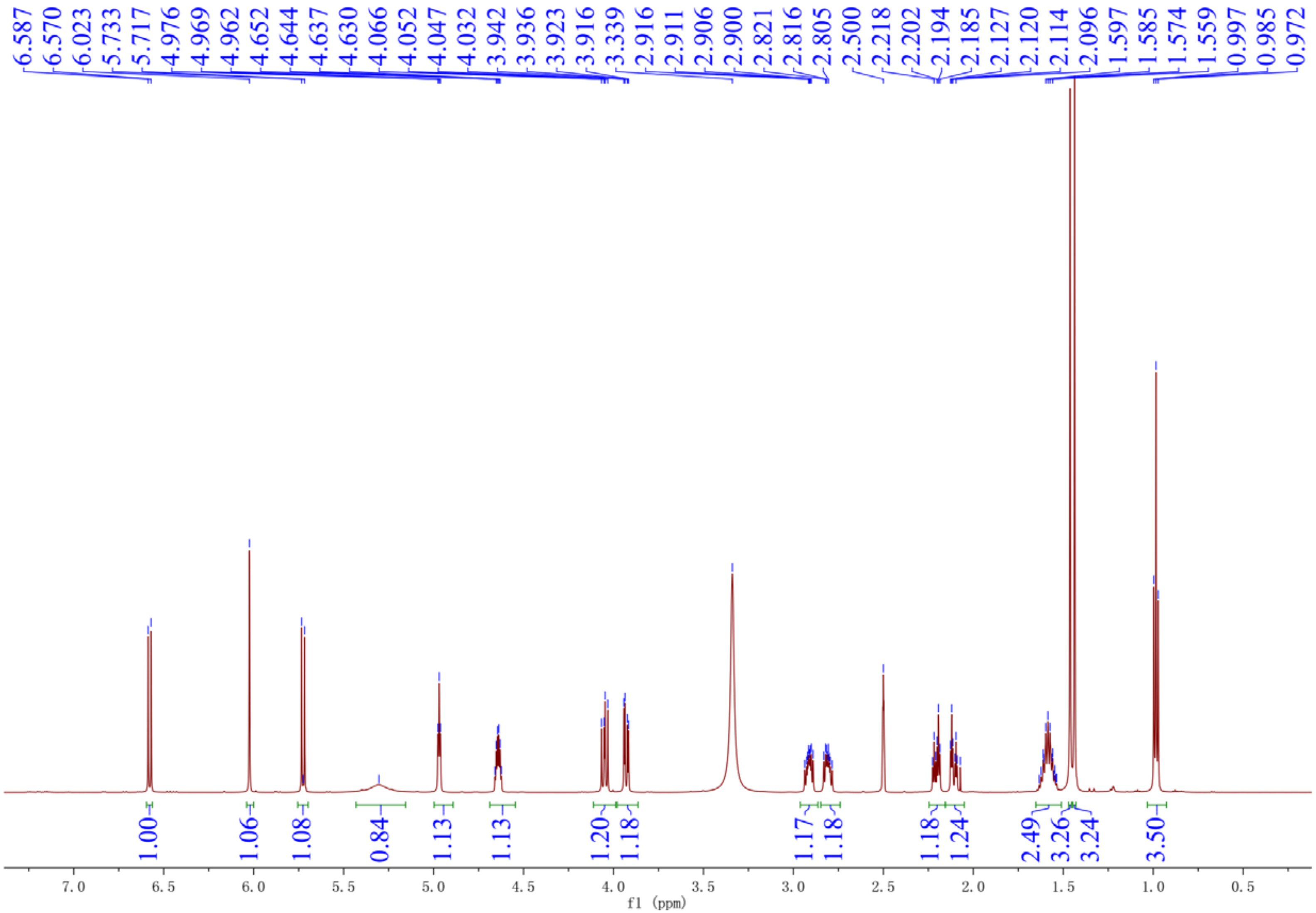
6.587
6.570
6.023
5.733
5.717
4.976
4.969
4.962
4.652
4.644
4.637
4.630
4.066
4.052
4.047
4.032
3.942
3.936
3.923
3.916
3.339
2.916
2.911
2.906
2.900
2.821
2.816
2.805
2.500
2.218
2.202
2.194
2.185
2.127
2.120
2.114
2.096
1.597
1.585
1.574
1.559
0.997
0.985
0.972
1.00
1.06
1.08
0.84
1.13
1.13
1.20
1.18
1.17
1.18
1.18
1.24
2.49
3.26
3.24
3.50
7.0
6.5
6.0
5.5
5.0
4.5
4.0
3.5
3.0
2.5
2.0
1.5
1.0
0.5
f1 (ppm)

28

F

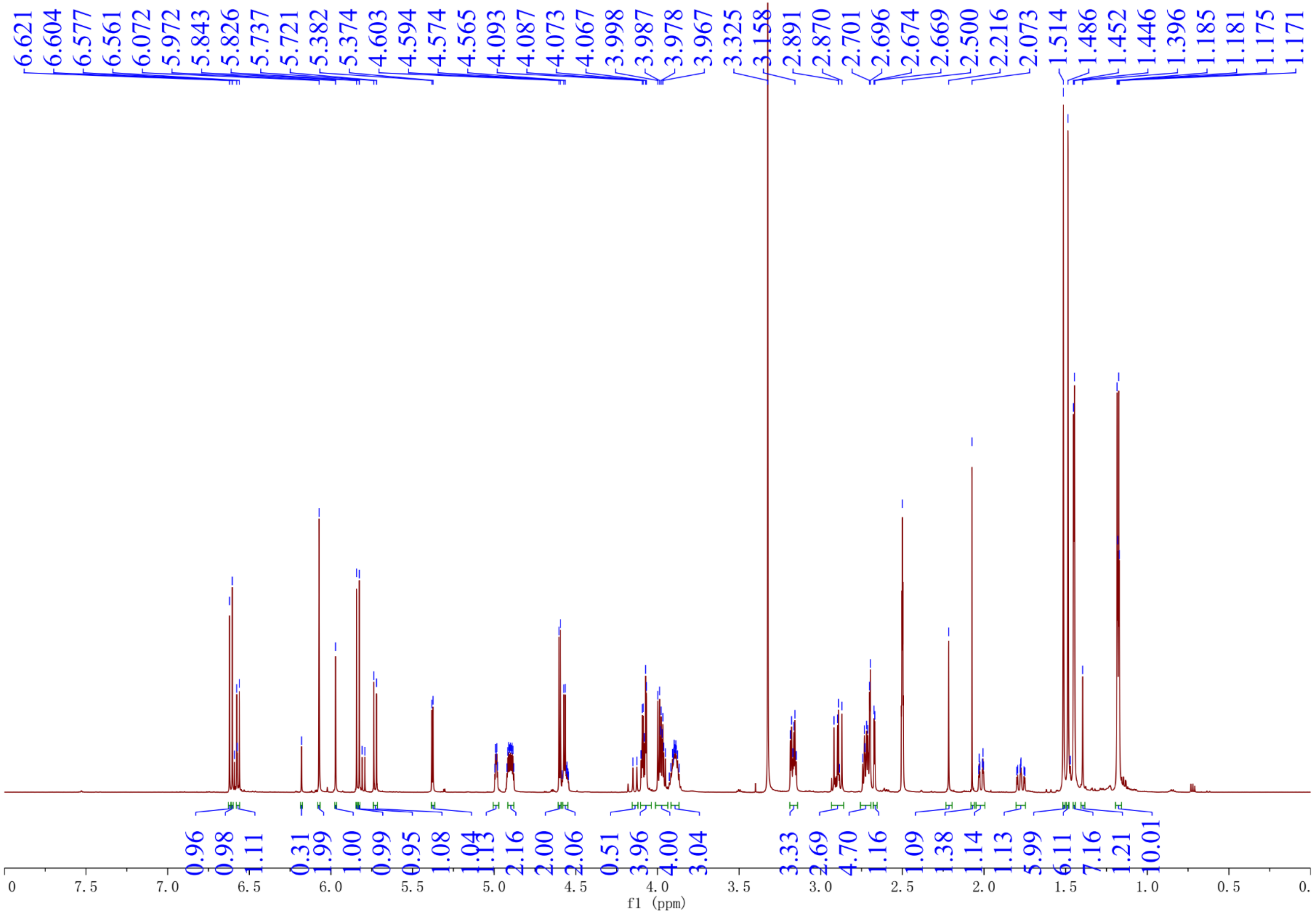

6.621
6.604
6.577
6.561
6.072
5.972
5.843
5.826
5.737
5.721
5.382
5.374
4.603
4.594
4.574
4.565
4.093
4.087
4.073
4.067
3.998
3.987
3.978
3.967
3.325
3.158
2.891
2.870
2.701
2.696
2.674
2.669
2.500
2.216
2.073
1.514
1.486
1.452
1.446
1.396
1.185
1.181
1.175
1.171
0.96
0.98
1.11
0.31
1.99
1.00
0.99
0.95
1.08
1.04
1.13
2.16
2.00
2.06
0.51
3.96
4.00
3.04
3.33
2.69
4.70
1.16
1.09
1.38
1.14
1.13
5.99
6.11
7.16
1.21
10.01
0 7.5 7.0 6.5 6.0 5.5 5.0 4.5 4.0 3.5 3.0 2.5 2.0 1.5 1.0 0.5 0.
f1 (ppm)

29

G

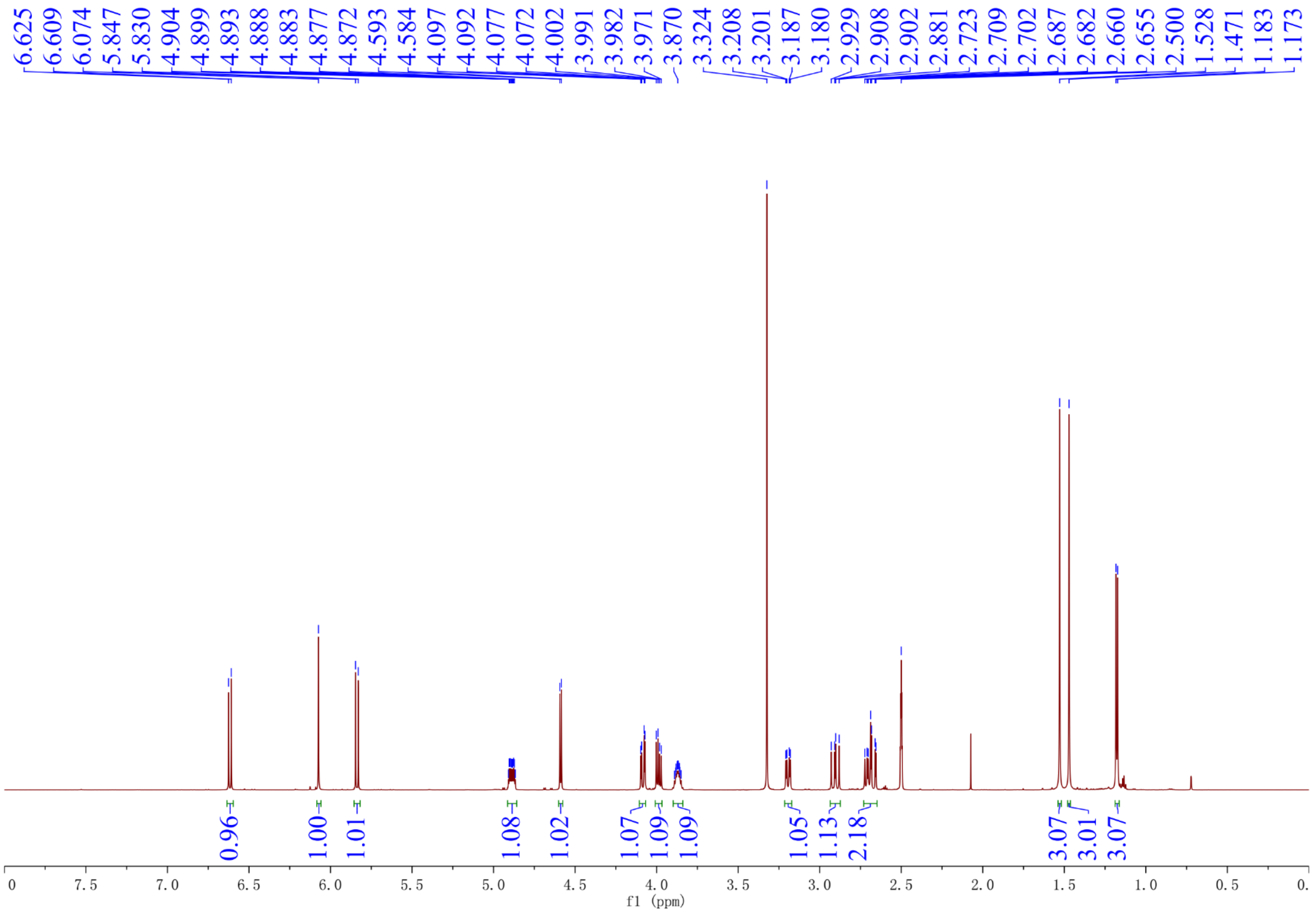

30

H

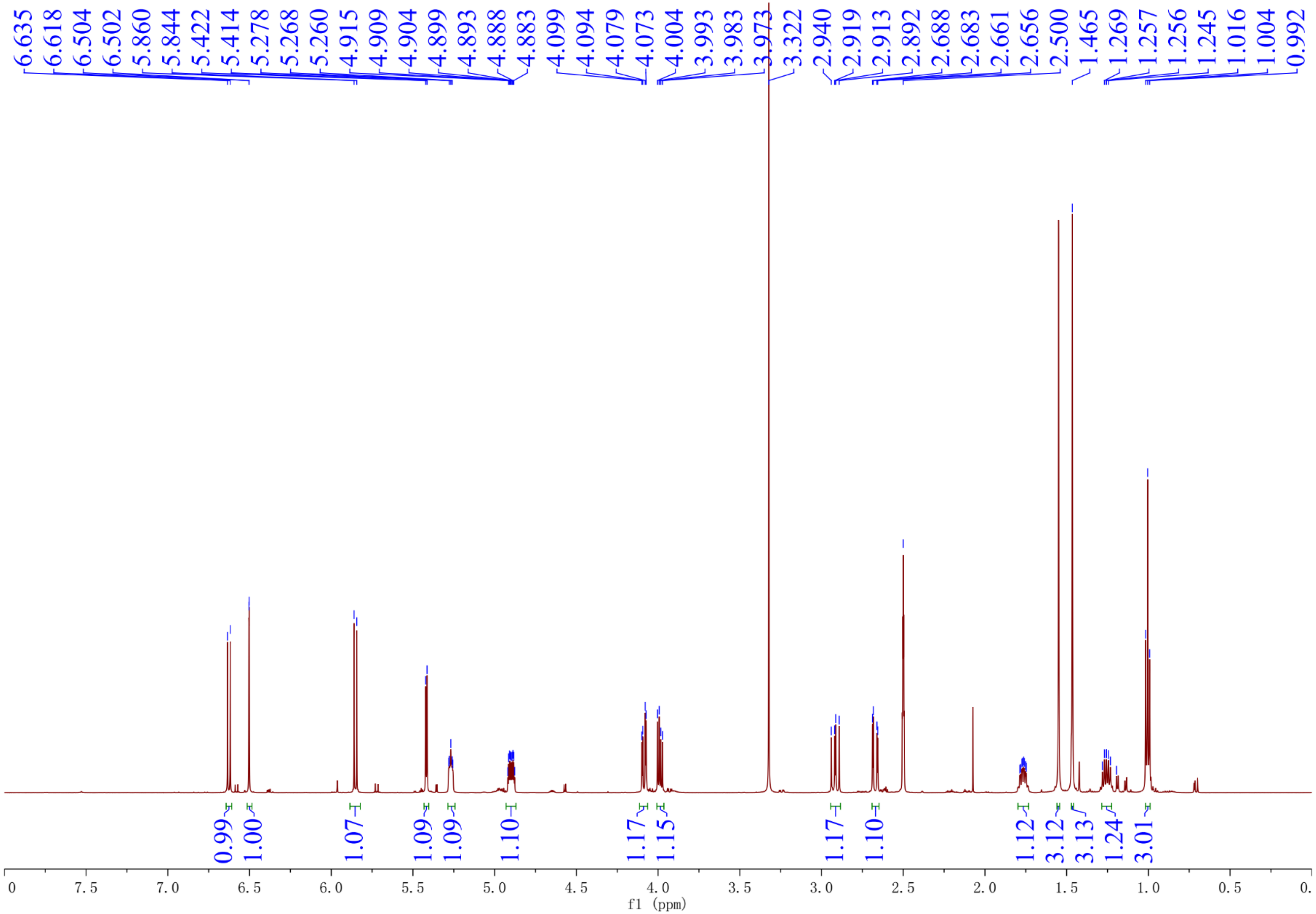

6.635
6.618
6.504
6.502
5.860
5.844
5.422
5.414
5.278
5.268
5.260
4.915
4.909
4.904
4.899
4.893
4.888
4.883
4.099
4.094
4.079
4.073
4.004
3.993
3.983
3.973
3.322
2.940
2.919
2.913
2.892
2.688
2.683
2.661
2.656
2.500
1.465
1.269
1.257
1.256
1.245
1.016
1.004
0.992
0.99
1.00
1.07
1.09
1.09
1.10
1.17
1.15
1.17
1.10
1.12
3.12
3.13
1.24
3.01
7.5
7.0
6.5
6.0
5.5
5.0
4.5
4.0
3.5
3.0
2.5
2.0
1.5
1.0
0.5
f1 (ppm)

31

I

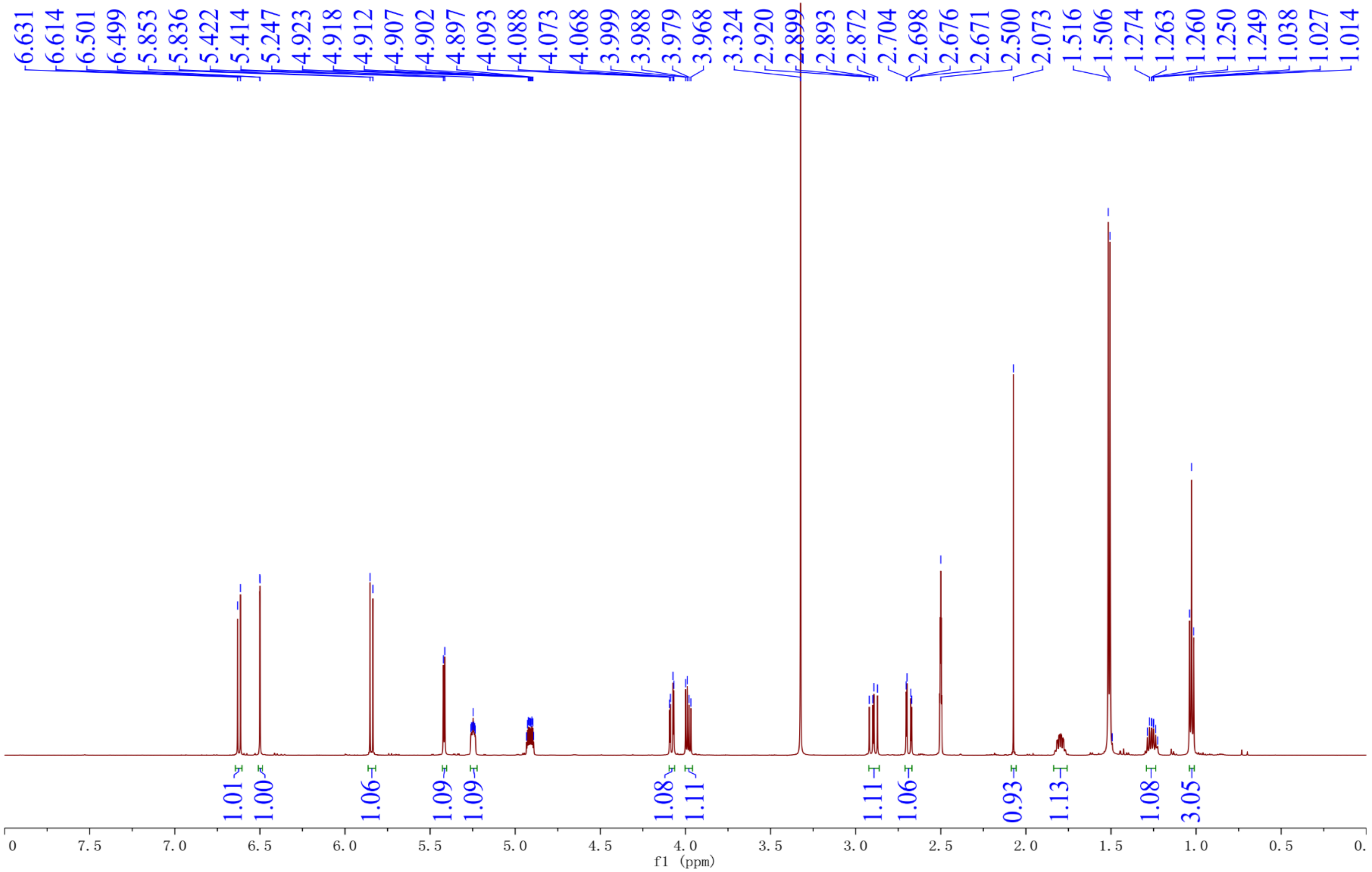
6.631
6.614
6.501
6.499
5.853
5.836
5.422
5.414
5.247
4.923
4.918
4.912
4.907
4.902
4.897
4.093
4.088
4.073
4.068
3.999
3.988
3.979
3.968
3.324
2.920
2.899
2.893
2.872
2.704
2.698
2.676
2.671
2.500
2.073
1.516
1.506
1.274
1.263
1.260
1.250
1.249
1.038
1.027
1.014
1.01
1.00
1.06
1.09
1.09
1.08
1.11
1.11
1.06
0.93
1.13
1.08
3.05
0
7.5
7.0
6.5
6.0
5.5
5.0
4.5
4.0
3.5
3.0
2.5
2.0
1.5
1.0
0.5
0.
f1 (ppm)

32

Supplemental figure S4 ^{1}H-NMR spectra of F18 and its metabolites. (A) F18; (B) M1; (C) M2-9; (D) M3-1; (E) 3-2; (F) M5-2; (G) M5-3; (H)
M
M5-4; (I) M5-5.
